# STEMIN transcription factor drives selective chromatin remodeling for gene activation within a relaxed chromatin during reprogramming in the moss *Physcomitrium patens*

**DOI:** 10.1111/tpj.70386

**Published:** 2025-08-01

**Authors:** Ruan Morné de Villiers, Gergo Palfalvi, Akinori Kanai, Yutaka Suzuki, Mitsuyasu Hasebe, Masaki Ishikawa

**Affiliations:** ^1^ Division of Evolutionary Biology National Institute for Basic Biology Okazaki 444‐8585 Japan; ^2^ Department of Basic Biology, School of Life Science SOKENDAI (The Graduate University for Advanced Studies) Okazaki 444‐8585 Japan; ^3^ Department of Comparative Development and Genetics Max Planck Institute for Plant Breeding Research Cologne Germany; ^4^ Department of Computational Biology and Medical Sciences the University of Tokyo Kashiwa 277‐8563 Japan

**Keywords:** reprogramming, stem cells, gene expression, chromatin accessibility, *Physcomitrium patens*, wounding, single‐cell analysis

## Abstract

Land plants exhibit remarkable cellular plasticity, readily reprogramming differentiated cells into stem cells in response to internal and external stimuli. While chromatin remodeling is crucial for cellular reprogramming, its interplay with gene expression during reprogramming into stem cells remains elusive. In the moss *Physcomitrium patens*, wounding induces reprogramming of leaf cells facing wounded cells to change into chloronema apical stem cells through the activation of the AP2/ERF transcription factor STEMIN. In this study, we employed multimodal single‐nuclei RNA and ATAC sequencing to explore the interplay between gene expression and chromatin dynamics during STEMIN‐mediated reprogramming. Profiling 20 883 single‐nuclei from gametophores, protonemata, and cut leaves, we identified 11 distinct cell types including reprogramming leaf cells. Our analysis revealed that reprogramming leaf cells exhibit a partly relaxed chromatin landscape and STEMIN transcription factors selectively enhance accessibility at specific genomic loci essential for stem cell formation. Thus, our results indicate that wounding initiates a broad chromatin relaxation, creating a permissive environment and specific transcription factors act to refine this permissive state by specifically relaxing chromatin regions critical for reprogramming.

## Significance Statement

Using single‐nucleus multiome analysis, this study reveals that wounding‐induced cellular reprogramming in the moss *Physcomitrium patens* involves widespread chromatin relaxation, establishing a permissive state for stem cell formation. Furthermore, STEMIN transcription factors selectively enhance chromatin accessibility at key loci, underscoring a hierarchical interplay between chromatin remodeling and gene activation during reprogramming.

## INTRODUCTION

Land plant and metazoan pluripotent stem cells have the ability to self‐renew and differentiate into various cell types to form tissues or organs (Greb & Lohmann, 2016; Heidstra & Sabatini, 2014). During differentiation, cells generated from stem cells acquire a new cell fate characterized by distinct gene expression patterns. Differentiated cells of plants can readily undergo reprogramming, in comparison to those of animals, highlighting the plasticity of plant cell identity (Ikeuchi et al., 2016; Ikeuchi et al., 2019; Wittmer & Heidstra, 2024). For instance, physical damage of a plant may trigger *de novo* formation of stem cell niches within mature tissues, enabling the regeneration of organs from wounded parts or the formation of an entire plant body from a dissected tissue segment (Ikeuchi et al., 2020; Liang et al., 2023). This cellular plasticity contributes to the environmental adaptability of plants, supporting their survival and enabling vegetative reproduction (Ishikawa & Hasebe, 2022; Umeda et al., 2021).

While, in effect, all cells in an organism share the same genomic sequence, they exhibit distinct gene expression patterns and specific cell fates during development, which are stably maintained thereafter (Chen & Dent, 2014; Ikeuchi et al., 2015). To this end, access to the genome for transcription is restricted. Genomic DNA wraps around histone proteins to form nucleosomes, further organized into euchromatin, an active state, or heterochromatin, a repressive state (Li et al., 2021). Epigenetic modifications, such as histone tail modifications and DNA methylation, regulate the packaging and expression of the genome within each cell, thereby determining cell identity and fate. Gene expression changes during cellular reprogramming should therefore be accompanied by the corresponding genetic loci becoming open or closed (Chen et al., 2023; Wang & Sung, 2024). Pioneer transcription factors, such as the Yamanaka factors that induce pluripotency in mammals (Takahashi & Yamanaka, 2006) and LEAFY (LFY) in flowering plants (Jin et al., 2021; Lai et al., 2021), play a key role in opening closed chromatin to allow binding by other factors (Barral & Zaret, 2024). Upon ectopic expression of these factors, somatic loci are rapidly silenced, while pluripotency loci are activated more gradually. Consequently, higher order chromatin structure is established in a stepwise manner; however, the underlying mechanisms remain unclear (Li et al., 2021).

With a more pliable genomic state achieved, what remains to be done is the establishment of a cell state predisposed for regeneration. Wound‐induced regeneration in land plants relies on the activation of key transcription factors to establish a new gene regulatory network (Ikeuchi et al., 2020; Liang et al., 2023). In shoots of *Arabidopsis thaliana*, upon wounding, *WOUND‐INDUCED DEDIFFERENTIATION* (*WIND*) genes, encoding an APETALA2 (AP2)/ETHYLENE‐RESPONSIVE TRANSCRIPTION FACTOR (ERF), are induced and function in the promotion of callus formation and shoot regeneration via direct induction of another AP2/ERF transcription factor *ENHANCER OF SHOOT REGENERATION 1* (*ESR1*: Iwase et al., 2011; Iwase et al., 2017; Iwase et al., 2021; Banno et al., 2001). In roots, the AP2/ERF transcription factor ETHYLENE RESPONSE FACTOR 115 (ERF115), induced by wounding, together with an accumulation of auxin, specifies stem cell identity (Canher et al., 2020). In addition to transcriptional activation, chromatin decondensation has been observed during various reprogramming processes, including protoplast regeneration (Chupeau et al., 2013; Ondřej et al., 2009; Tessadori et al., 2007; Zhao et al., 2001), shoot regeneration from epidermal cells in *Torenia fournieri* (Morinaka et al., 2021), and cellular reprogramming of leaf cells into stem cells in mosses (Sato et al., 2017). These observations suggest that cellular reprogramming across land plants is associated with changes in chromatin accessibility. However, the relationship between the activity of reprogramming‐associated transcription factors and chromatin remodeling remains unclear, particularly how localized chromatin modifications relate to the broader chromatin changes observed during reprogramming.

The moss *Physcomitrium patens* exhibits a unique capability wherein differentiated cells can directly change into stem cells (Ishikawa et al., 2011; Ishikawa & Hasebe, 2022). When leaves are detached from gametophores, most of the leaf cells facing the cut undergo cellular reprogramming into chloronema apical stem cells, exhibiting tip growth and cell division to regenerate chloronemata without the need for exogenous plant hormones, while the leaf cells not facing the cut retain their original morphological state. During this process, the expression of the *STEM CELL‐INDUCING FACTOR 1* (*STEMIN1*) gene and its two paralogous genes *STEMIN2* and *STEMIN3*, which all encode AP2/ERF transcription factors, is activated within leaf cells that face the cut before tip growth and cell division (Ishikawa et al., 2019). Deletion in all three *STEMIN* genes delays the formation of chloronema apical stem cells, while the ectopic induction of STEMIN1 changes local histone modifications of its direct target genes and induces cellular changes from leaf cells to chloronema apical stem cells (Ishikawa et al., 2019). Thus, STEMINs function within an intrinsic mechanism underlying reprogramming in response to wounding.

While previous studies have investigated gene expression changes during *P. patens* reprogramming using bulk transcriptomics (Nishiyama et al., 2012) and single‐cell approaches (Chen et al., 2024; Hata et al., 2025; Kubo et al., 2019), the interplay between gene expression and chromatin dynamics during this process remains to be elucidated. In this study, we explored the interplay between gene expression and chromatin dynamics during STEMIN‐mediated reprogramming triggered by wounding in *P. patens* using a multimodal approach that integrated single‐nuclei RNA sequencing (snRNA‐seq) and single‐nuclei Assay for Transposase‐Accessible Chromatin sequencing (snATAC‐seq). We profiled 20 883 single nuclei from gametophores, protonemata, and cut leaves and identified 11 distinct cell types, including reprogramming leaf cells. Our analysis revealed a correlation between chromatin accessibility and gene expression across all differentiated cells and stem cells, while in the reprogramming leaf cells, this correlation was significantly weaker. Our snATAC‐seq data further demonstrated a more relaxed chromatin state in reprogramming leaf cells, suggesting that wounding induces genome‐wide chromatin structural changes. Crucially, we found that STEMIN selectively loosens chromatin at specific gene loci within this relaxed environment, activating genes required for the leaf‐to‐stem‐cell transition. These findings suggest that STEMIN transcription factors promote gene expression through a multifaceted mechanism, encompassing both local histone modifications and selective chromatin relaxation around specific genomic loci within a permissive chromatin environment. Our study highlights the dynamic nature of chromatin accessibility during wound‐induced reprogramming and provides insight into how wounding reshapes overall genome architecture.

## RESULTS

### Identification of reprogramming leaf cells by cell clustering

**Figure 1:**
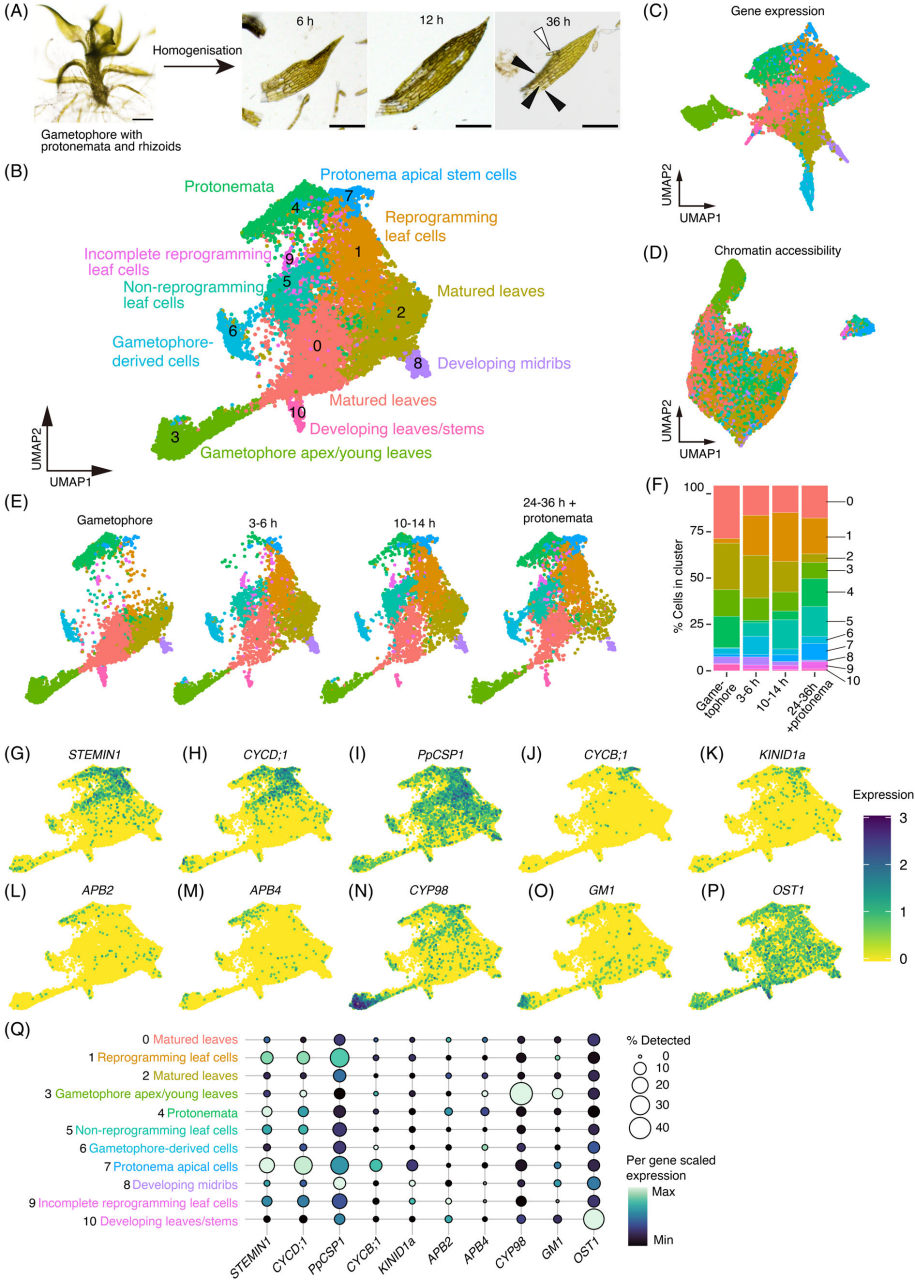
Clustering of the combined snRNA‐seq and snATAC‐seq of protonemata, gametophores, and cut leaves. (A) Representative brightfield images of a gametophore with protonemata and rhizoids, and cut gametophore leaves at different time points (6, 12, and 36 h) after cutting with a homogenizer. Black and white arrowheads indicate chloronema apical stem cells formed from leaf cells and fragmented protonemata, respectively. Scale bars: 100 μm. (B–D) Two‐dimensional UMAP embedding of nuclei from all samples based on the combined data from RNA‐seq and ATAC‐seq data (B), RNA‐seq data (C), and ATAC‐seq data (D). The UMAP plots display 11 cell clusters (0–10) with distinct colors based on the combined RNA and ATAC clustering shown in (B). (E) UMAP of combined data from wild‐type and ∆stemin plants split by timepoints (gametophores, 3–6 h, 10–14 h, and 24–36 h + protonemata). (F) Stacked bar graphs showing the percentage distribution of cells within each cluster over time, as visualized in (c). (G–P) UMAP visualization of the representative genes for reprogramming and specific developmental stages. *STEMIN1*, *CYCD;1* and *PpCSP1* for reprogramming leaf cells, *CYCB;1* and *KINID1a* for protonema apical stem cells; *APB2* and *APB4* for protonema cells; *CYP98* and *GM1* for gametophore apex/young leaves; and *OST1* for apical leaves and gametophore stems. The color bar denotes the relative expression level. (Q) Dot plot showing the marker genes visualized in (G–Q). Dot diameters and colors denote the proportion of cells and the scaled average expression levels of the genes, respectively. Expression is scaled to the minimum and maximum cluster averages per gene of interest.

To investigate the relationship between gene expression and chromatin accessibility in STEMIN‐mediated reprogramming following wounding, we collected cut leaves from both the wild‐type and a triple *STEMIN*‐deletion mutant plants (∆stemin1∆stemin2∆stemin3#6–48‐1 [Gu et al., 2020]; hereafter referred to as ∆stemin) over three time intervals: 3–6 h, 10–14 h, and 24–36 h after tissue disruption, along with untreated gametophores (Figure 1a). Following nuclei isolation and fluorescence‐activated nuclei sorting, equal numbers of nuclei within the designated time window were pooled to generate heterogeneous nuclei samples. This approach aimed to capture variations occurring with smaller time frames rather than restricting the six samples to fixed time points after tissue disruption. Additionally, we pooled nuclei from cut leaves 24 and 36 hurs after cut with those from 1‐week‐old protonemata (24–36 h + protonemata). The sorted nuclei were used for the generation of cDNA and ATAC‐seq libraries using the 10x Genomics Chromium system (see Materials and Methods for details). Sequenced libraries were processed using the Cellranger‐ARC pipeline (10x Genomics). Additional quality thresholds were enforced (Figure S1), yielding a total of 20 883 high‐quality nuclei from wild‐type and ∆stemin mutant samples, which were used to construct the final multiomic atlas (Table S1).

To process the count data from all eight samples, we used Seurat v4 (Hao et al., 2021) for the RNA and Signac (Stuart et al., 2021) for the ATAC modalities, then merged using weighted nearest neighbors (wnn). Batch effects were removed using Harmony (Korsunsky et al., 2019). Dimensionality reduction using Uniform Manifold Approximation and Projection (UMAP) and clustering were first performed on the wnn graph (Figure 1B).

We obtained 11 distinct cell‐type clusters (0–10) at a resolution of 0.4, as visualized by UMAP (Figure 1B; Figure S2). Superimposition of the clustering scheme on the individual gene expression and chromatin accessibility UMAPs distinctly indicated that the multimodal clustering is most consistent with the gene expression modality (Figure 1C, D), while some of the clusters are less distinct when only the chromatin accessibility data are considered (Figure 1D).

We next split the UMAP visualizations for each time point to evaluate them individually (Figure 1E). In gametophore samples containing protonemata, cells within clusters 1, 5, 7, and 9 were either barely detectable or present in low numbers, while these populations increased substantially following tissue disruption, at 3–6 h and 10–14 h, suggesting that these clusters predominantly consist of cell types induced by leaf cutting (Figure 1E,F). To clarify the cellular identities within these clusters, we examined the expression patterns of known reprogramming‐related genes in the data. *STEMIN1* and its direct target gene *CYCLIN D;1* (*CYCD;1*) were detected in clusters 1 and 7, with higher expression levels compared with other clusters (Figure 1G, H, Q). In contrast, expression levels of these genes were detectable, but at lower levels in clusters 5 and 9. Similarly, *P. patens COLD‐SHOCK DOMAIN PROTEIN 1* (*PpCSP1*), encoding an RNA‐binding protein with a positive role in reprogramming (Li et al., 2017), exhibited high expression in clusters 1 and 7 (Figure 1I, Q).

Among these clusters, cluster 7 was further distinguished by the expression of *CYCLIN B1* (*CYCB;1*: Ishikawa et al., 2011) and *KINESIN FOR INTERDIGITATED MICROTUBULES a1* (*KINID1a*: Hiwatashi et al., 2008), which function in the G2 to M phase of the cell cycle and cytokinesis, respectively (Figure 1J, K, Q). CYCB1‐GUS lines, which express a translational fusion protein of CYCB1 and β‐glucuronidase (GUS) driven by its native promoter, showed GUS activity in chloronema and caulonema apical stem cells (collectively referred to as protonema apical stem cells), leaf primordia, and leaf cells specifically at 48 h after cutting but not 24 h (Figure S3). *KINID1a* is also expressed at protonema apical stem cells (Hiwatashi et al., 2008). These results suggest that cluster 1 primarily comprises reprogramming leaf cells in a pre‐division stage, whereas cluster 7 represents protonema apical stem cells undergoing cell division, either derived from existing protonemata or regenerated from cut leaves.

### Characterization of cell clusters based on gene expression patterns

Cells in clusters 5 and 9, potentially induced by leaf cutting, exhibited lower expression of *STEMIN1*, *CYCD;1*, and *PpCSP1* compared with clusters 1 and 7. However, cluster 9 showed higher expression of these genes than cluster 5 and consistently represented a smaller cell population than cluster 5 at both 3–6 h and 10–14 h post‐disruption. Given that approximately 30% of leaf cells in cut leaves undergo successful reprogramming, while the remaining 70% do not (Ishikawa et al., 2011), the larger size of cluster 5 compared with cluster 9 suggests that cluster 5 likely represents non‐reprogramming leaf cells. On the other hand, since a subset of cells facing cut in cut leaves transiently activates the *STEMIN1* promoter but fails to exhibit tip growth and subsequently loses promoter activity (Ishikawa et al., 2019), cluster 9 may represent incompletely reprogrammed cells that face the cut edge where *STEMIN* expression is initiated but prematurely terminated, preventing complete reprogramming (Figure 1D). Further investigation of the spatial expression variability within clusters 5 and 9 is warranted and will be the focus of future studies.

Cells in clusters 0, 2, 3, and 4 were predominantly found in gametophore samples including protonemata (Figure 1E,F). *AINTEGUMENTA, PLETHORA*, *and BABY BOOM 2* (*APB2*) and *APB4* genes, localized in protonema cells and emerging gametophore apical stem cells, but not in protonema apical stem cells (Aoyama et al., 2012), were enriched in cluster 4 (Figure 1L, M, Q), suggesting that cluster 4 likely represents cells derived from protonemata. Consequently, the remaining clusters, 0, 2, and 3, were likely to represent cells derived from gametophores. To further characterize specific cell types within other clusters, we examined the expression of genes associated with gametophore development across the data. *Cytochrome P450 monooxygenase 98* (*CYP98*; Renault et al., 2017) and *GAMETOPHORE MARKER1* (*GM1* [Pp3c21_6170]; Hata et al., 2025) are genes whose promoters are activated in the apices and young leaves of gametophores, but not in protonemata. These genes were enriched in cluster 3 (Figure 1N, O, Q), suggesting that the cells in this cluster originate from young gametophore tissue. In contrast, clusters 0 and 2 appear to primarily consist of cells derived from fully developed gametophore leaves.

An *Arabidopsis thaliana OPEN STOMATA* homologue, *PpOST1*, expressed in gametophore stems and developing leaves but not in matured leaves (Chen et al., 2024), showed elevated expression in cluster 10 (Figure 1P,Q), suggesting that cluster 10 represents cells from young gametophore tissue including young leaves as cluster 3. In cluster 8, we found high expression of *P. patens VND*‐, *NST/SND*‐, *SMB*‐*RELATED PROTEIN 1* (*PpVNS1*), *PpVNS2*, *PpVNS5*, and *PpVNS8* genes, which are known to be commonly expressed in both midribs and rhizoids (Xu et al., 2014), suggesting that this cluster originates from either tissue. However, the homeodomain‐leucine zipper I gene *PpHB7*, which is predominantly expressed and functions in rhizoids (Sakakibara et al., 2003), was not detected in cluster 8 (Figure S4). This argues against a rhizoid origin and instead supports the midrib as the source of cluster 8. For cluster 6, we could not identify any marker genes. Nonetheless, we provisionally assigned it as gametophore‐derived cells (Figure 1c), based on its exclusive proximity to cluster 2, which we annotated as matured leaves.

Previously, we established a single‐cell digital gene expression (1cell‐DGE) method, which uses micromanipulation to extract nuclei of individual living cells from intact tissue, and identified differentially expressed genes (DEGs) in reprogramming leaf cells at 0 and 24 h after excision (Kubo et al., 2019). We therefore examined the expression of the top 10 DEGs identified at each time point using 1cell‐DGE. Consistently, the top 10 DEGs at 0 h were highly expressed in clusters 0 and 2, corresponding to mature leaves, and showed low expression in cluster 1, representing reprogramming leaf cells (Figure S6). Conversely, the top 10 DEGs at 24 h exhibited high expression in cluster 1 and low expression in clusters 0 and 2. These consistent expression patterns support the reliability of our current multimodal analysis. Thus, we identified 11 distinct clusters, including reprogramming leaf cells (Figure 1D). While spatiotemporal expression analysis of additional marker genes (Figure S5) could offer valuable insights into their cellular origins, further investigation is beyond the scope of this study, which focuses on reprogramming. This analysis should be prioritized in future research.

### Comparison of reprogramming‐related gene expression between wild‐type and ∆stemin plants

**Figure 2:**
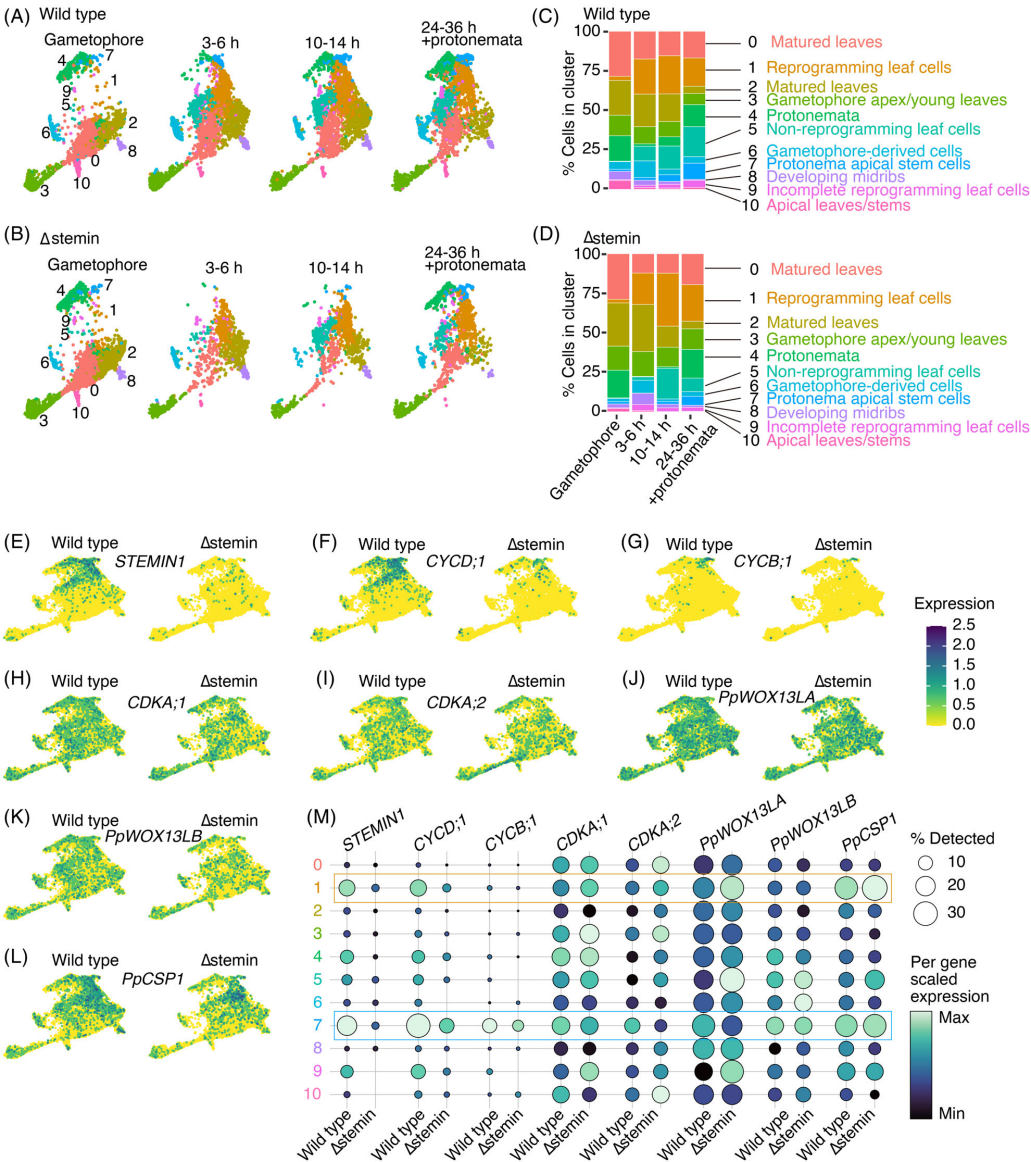
Comparison of reprogramming‐related gene expression between wild‐type and ∆stemin. (A, B) UMAP of wild‐type (A) and ∆stemin (B) plants by timepoints (gametophore, 3–6 h, 10–14 h, and 24–36 h + protonemata). The UMAP plot displays 11 cell clusters (0–10) with distinct colors as in Figure 1. (C, D) Stacked bar graphs showing the distribution of cells within each cluster for individual time points in wild‐type (A) and ∆stemin (B) plants. (E–L) Visualization of reprogramming‐related gene expression on UMAP of wild‐type (left) and ∆stemin (right) plants, combined across four different time points. *STEMIN1* (E), *CYCD;1* (F), *CYCB;1* (G), *CDKA;1* (H), *CDKA;2* (I), *PpWOX13LA* (J), *PpWOX13LB* (K), and *PpCSP1* (L). (M) Dot plot showing the reprogramming‐related genes visualized in (E–L). Dot diameters and colors denote the proportion of cells and the average expression levels of the genes per cluster, respectively. Expression is scaled to the minimum and maximum cluster averages per gene of interest.

The reactivation of cell division is essential for the regeneration of damaged tissues, a fundamental process shared by all multicellular organisms. Therefore, to compare the expression of cell cycle‐related genes between wild‐type and ∆stemin plants over time, we divided the UMAP visualizations between each genotype and across different time points (Figure 2A,B). Reprogramming leaf cells (cluster 1) increased proportionately after tissue disruption in both wild‐type and ∆stemin samples (Figure 2A–D), indicating that wounding initiated the reprogramming process in both lines. On the other hand, in wild‐type plants, *STEMIN1* and *CYCD;1* were expressed in cells within the reprogramming cluster, but were repressed in ∆stemin plants (Figure 2E,F,M). Additionally, *CYCB;1* was downregulated in the protonema apical stem cells (cluster 7) of ∆stemin plants compared with that of wild‐type (Figure 2G,M), while the expression of *CYCLIN‐DEPENDENT KINASE A;1* (*CDKA;1*) and *CDKA;2* genes (Ishikawa et al., 2011) was slightly higher in reprogramming leaf cells of ∆stemin compared with those of the wild‐type (Figure 2H, I, M). Since CDKA activation through interaction with CYCD eventuates progression of the cell cycle, these results indicate that wound‐activated *STEMIN* genes are necessary for driving cell cycle re‐entry and progression through the regulation of CDKA activity.

We have previously proposed that the STEMIN‐mediated pathway functions in parallel to the *P. patens* WUSCHEL‐related homeobox 13‐like (PpWOX13L) transcription factor pathway, the PpCSP pathway, and the autophagy pathway (Ishikawa & Hasebe, 2022). *PpWOX13L* genes play a crucial role in tip growth during wound‐induced cellular reprogramming by regulating the expression of genes encoding cell wall modification enzymes, including B‐type EXPANSIN4 (EXPB4) (Sakakibara et al., 2014). Our analysis revealed that *PpWOX13LA* expression was higher in reprogramming leaf cells of Δstemin compared with the wild‐type, whereas *PpWOX13LB* showed no difference in expression between wild‐type and Δstemin plants in both reprogramming leaf cells and protonema apical stem cells (Figure 2J, K, M). Additionally, *PpCSP1* was upregulated in the reprogramming cells in the Δstemin plant but remained unchanged in the protonema apical stem cells (Figure 2L, M). These findings suggest that the PpWOX13L and PpCSP pathways remain functional during reprogramming in the absence of STEMINs. In contrast, we observed a general increase of several autophagy‐related genes in reprogramming leaf cells of Δstemin plants (Figure S7), suggesting a suppression of autophagy activity by STEMINs.

### STEMIN‐mediated gene expression changes in reprogramming cells

**Figure 3:**
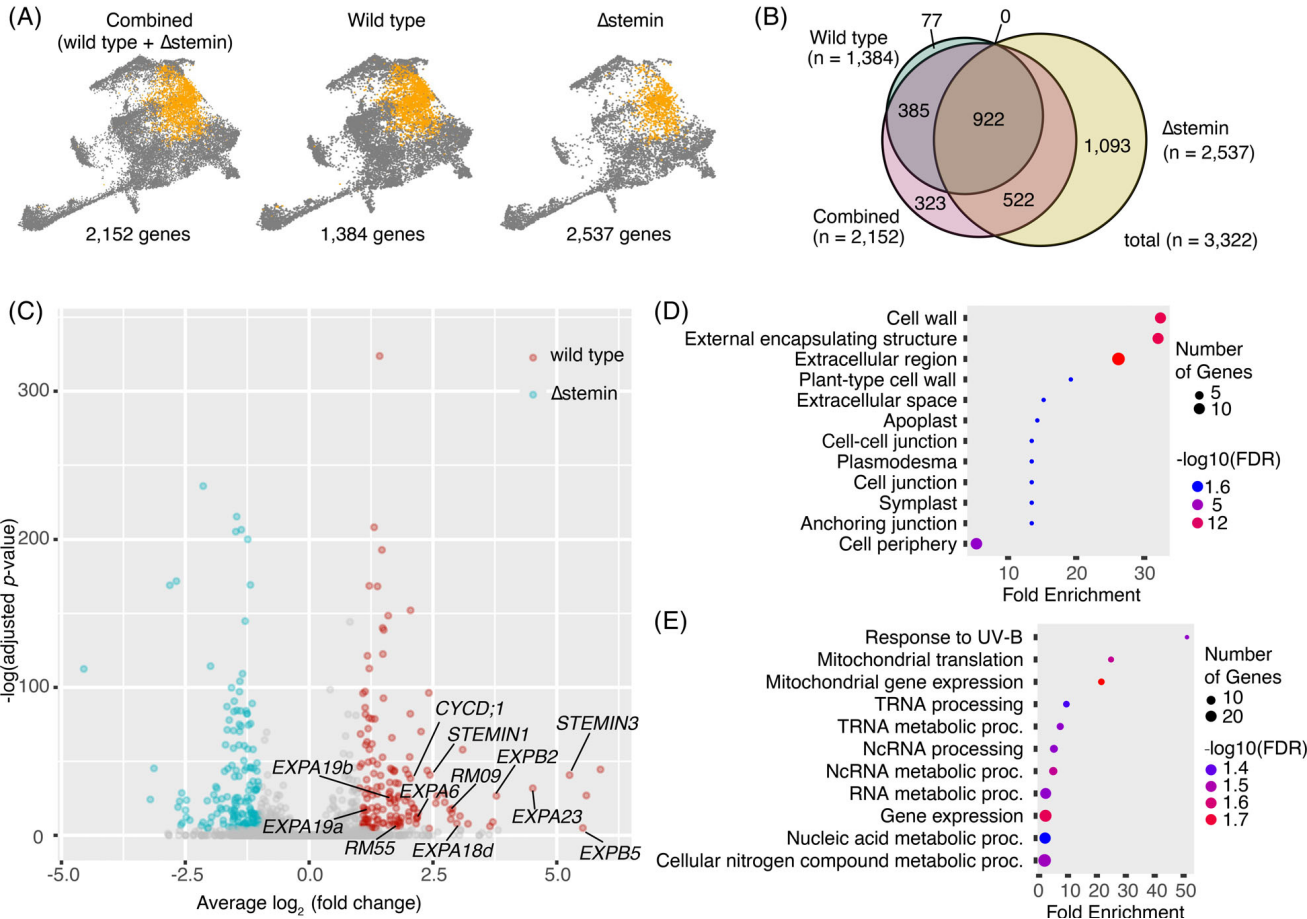
Identification of differentially expressed genes between wild‐type and ∆stemin reprogramming cells. (A) UMAP visualization of reprogramming cell cluster in combined wild‐type and ∆stemin samples (left), along with individual wild‐type (middle) and ∆stemin (right) samples. The number of DEGs found in the cells within that cluster of those samples is shown below each UMAP. (B) Venn diagram showing the overlap of differentially expressed genes between combined WT and ∆stemin samples, and individual WT and ∆stemin samples. (C) Volcano plot showing differentially expressed genes between wild‐type and ∆stemin reprogramming cell clusters. Blue and red dots indicate significantly upregulated genes in WT and ∆stemin samples, respectively (adjusted *P*‐value <0.01, average log2(fold change) >1 or <−1). Gray dots indicate no significant difference in gene expression between wild‐type and ∆stemin samples. *STEMIN1*, *STEMIN3*, *CYCD;1*, *EXPANSIN*s (*EXPA6*, *EXPA18d*, *EXPA19a*, *EXPA19b*, *EXPA23*, *EXPB2*, *EXPB5*), *RM09*, and *RM55* genes, which are significantly upregulated specifically in wild‐type samples, are highlighted on the volcano plot. (D, E) GO enrichment analyses of significantly upregulated genes in wild‐type (D) and ∆stemin (E) samples. Gene ontology enrichment analyses were performed using ShinyGO v0.81 (http://bioinformatics.sdstate.edu/go/). The enriched GO biological processes of significantly upregulated genes are indicated (FDR <0.05).

Next, we identified DEGs between the reprogramming cluster and the remaining cells using a Wilcoxon rank sum test, with a *P*‐value cutoff of 0.01, implemented through Seurat's FindMarkers function (Stuart et al., 2021). Of the 26 964 genes detected by the snRNA‐seq, 2152 genes showed differential expression in the reprogramming cluster when all samples were combined (Figure 3A; Table S2). When samples were grouped by genotype, 1384 and 2537 genes were differentially expressed in the wild‐type and in the ∆stemin plants, respectively (Figure 3A; Table S2). In total, 3322 unique DEGs were identified after accounting for overlaps between gene sets (Figure 3B). These genes were classified as either upregulated (log2‐fold change >1) or downregulated (log2‐fold change <−1), with 1775 upregulated and 1547 downregulated genes.

To identify genes specifically upregulated in wild‐type and ∆stemin reprogramming cell clusters, we conducted an additional Wilcoxon rank sum test limiting the genes available to test to the 1775 upregulated genes. This analysis compared expression levels between wild‐type and ∆stemin genotypes in the 3585 cells within the reprogramming cluster. We found that 54 genes were significantly upregulated in wild‐type reprogramming cells, while 108 genes were upregulated in ∆stemin mutant cells (Figure 3C; Table S3). The 54 genes specifically upregulated in wild‐type reprogramming cells included *EXP* genes and genes encoding cell wall modification enzymes, xyloglucan:xyloglucosyl transferase (XTH), in addition to the *CYCD;1* gene (Figure 3C; Table S3).

Gene ontology (GO) enrichment analysis of upregulated genes in wild‐type samples revealed a significant enrichment of genes involved in cell wall, external encapsulating structure, and extracellular region (Figure 3D), suggesting that STEMIN plays a crucial role in regulating cell wall remodeling during wound‐induced reprogramming. Additionally, we found that 15 of the 54 genes were also upregulated by *PpWOX13L* genes (Sakakibara et al., 2014). We did not detect a decrease in the expression of either *PpWOX13LA* or *PpWOX13LB* genes in ∆stemin samples (Figure 2J, K, M), suggesting that the activation of cell wall loosening genes is co‐regulated by both *PpWOX13L* and *STEMIN* genes (Figure S8). Given that *A. thaliana* WIND1 activates the PpWOX13L ortholog AtWOX13 to drive cell wall loosening gene expression (Ikeuchi et al., 2022; Iwase et al., 2011; Ogura et al., 2023), the co‐regulation of these genes by PpWOX13L and STEMIN in *P. patens* suggests a conserved role of AP2/ERF‐WOX13 for coordinating cell wall remodeling during wound‐induced reprogramming in land plants. On the other hand, GO analysis of upregulated genes in ∆stemin plants identified an enrichment of RNA processing‐related functions, implying repression of those genes by STEMINs during reprogramming (Figure 3e).

Of the 54 genes specifically upregulated in wild‐type reprogramming cells, 15 were previously identified as directly upregulated targets of STEMIN1 (Ishikawa et al., 2019; Table S3), suggesting direct activation by STEMIN1 upon wounding (Figure S9). The remaining genes are likely regulated by STEMIN2, STEMIN3, or transcription factors downstream of STEMINs. In contrast, of the 108 genes specifically upregulated in ∆stemin reprogramming cells (i.e., repressed by STEMINs after wounding), 11 were directly upregulated by STEMIN1 induction (Ishikawa et al., 2019). This implies that while STEMIN1 is able to activate these 11 genes, STEMIN2 and/or STEMIN3 may subsequently repress their expression (Figure S9). Alternatively, STEMIN1 overexpression in a context lacking tissue damage, and by extension, without the permissive chromatin state or the co‐factors like PpWOX13L, may trigger irregular gene expression patterns.

### Links between gene expression and chromatin accessibility during reprogramming

**Figure 4:**
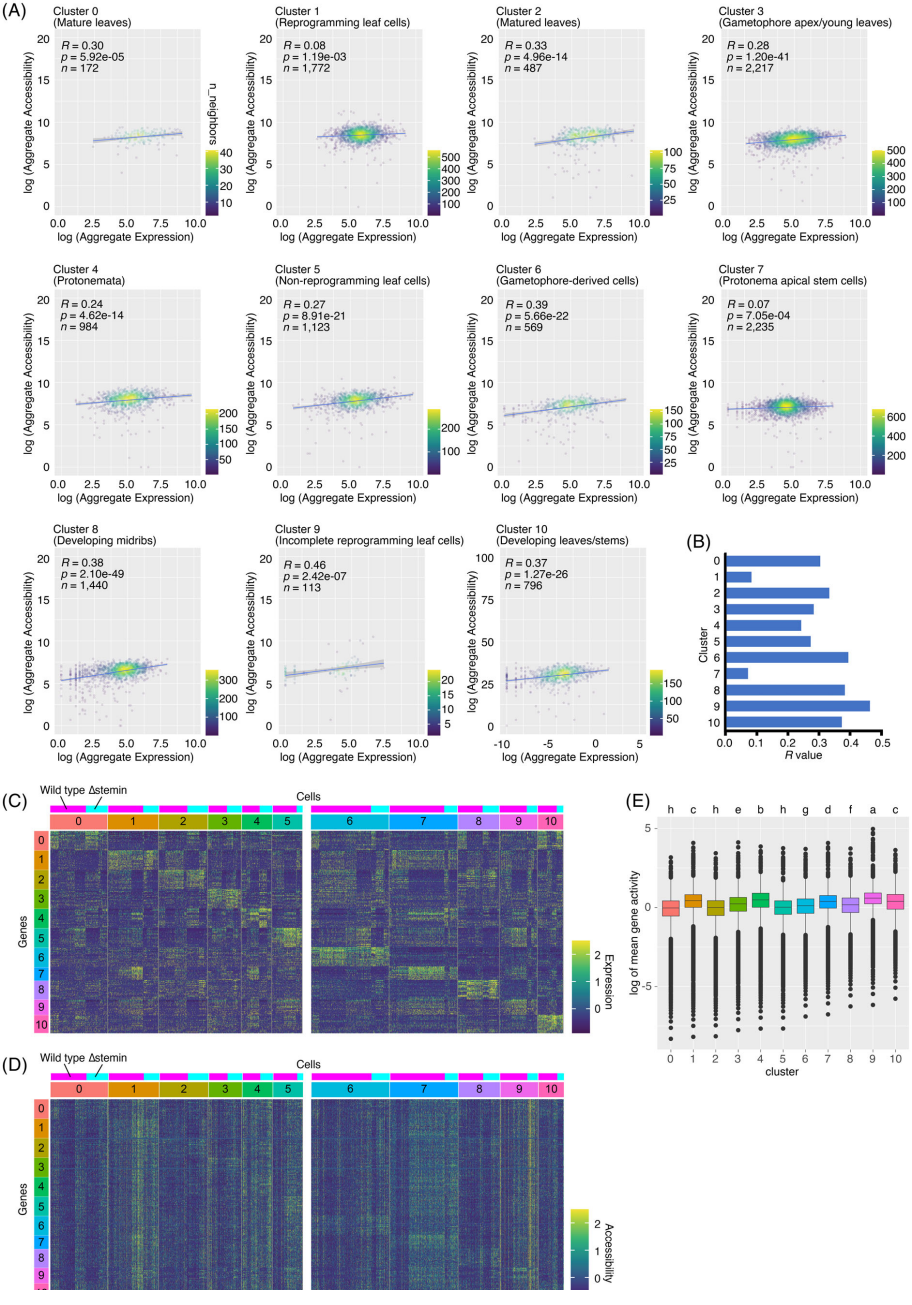
Correlation between gene expression and chromatin accessibility. (A) Scatter plots illustrating the Spearman's correlation between gene expression and chromatin accessibility for differentially expressed genes by cluster. Color represents density based on the number of nearest neighbors. The *X*‐axis corresponds to the sum of gene expression scores; the *Y*‐axis represents chromatin accessibility scores, calculated for each upregulated gene across cluster‐specified cells. (B) Comparison of Spearman's correlation coefficients between clusters. (C, D) Heatmaps displaying gene expression (C) and chromatin accessibility (D) of the top 10 ranked differentially expressed genes (DEGs) in each cluster. The color bars denote the relative expression level (C) and chromatin accessibility (D). (E) Comparison of chromatin accessibility in all genes within each cluster. Lowercase letters indicate significant differences (one‐way ANOVA and Tukey HSD test, *P* < 0.05).

To investigate the relationship between transcriptional activity and chromatin accessibility, we calculated Spearman's correlation coefficients for all expressed genes in each cluster of a combined wild‐type and ∆stemin sample set. Across all clusters, a weak positive correlation was observed, with *R* values ranging from 0.14 to 0.22 (Figure S10). In contrast, when analyzing only DEGs, identified as those exhibiting significant expression changes in each cluster relative to the remaining clusters (Figure 4A, B), reprogramming leaf cells (cluster 1) and protonema apical stem cells (cluster 7) exhibited negligible correlation (*R* = 0.08), whereas other clusters displayed a weak positive correlation (*R* = 0.23–0.38: Figure 4A, B). This suggests a weaker association between gene expression and chromatin accessibility specifically for genes undergoing expression changes during reprogramming and stem cell fate determination or maintenance, compared with other cell types.

To further explore this relationship, we selected the top 10 ranked genes exhibiting the highest expression levels within each cluster of wild‐type and ∆stemin plants (Figure 4C). We then analyzed the chromatin accessibility of their coding regions and 2‐kb flanking regions, including 2 kb upstream of the start codons (5′‐flanking) and 2‐kb downstream of the stop codons (3′‐flanking) (Figure 4D). A comparative analysis of gene expression and chromatin accessibility across different cell clusters revealed that genes specifically upregulated within individual clusters have their chromatin accessibility slightly increased compared with the other clusters (Figure 4D), supporting a weak correlation between their expression levels and the accessibility of their regulatory regions. On the other hand, in clusters 1, 4, 7, and 9, chromatin accessibility was elevated not only at regions corresponding to expressed genes but also at regions corresponding to genes that were not expressed in these clusters compared with other cell types (Figure 4D). This suggests that these clusters exhibit a broader increase in chromatin accessibility, regardless of gene expression level.

To explore genome‐wide changes in chromatin accessibility, we calculated the average ATAC‐seq read distribution in each gene body from the transcriptional start site (TSS) and transcriptional termination site (TTS) for each nucleus within individual cell clusters. The analysis revealed increased chromatin accessibility in clusters 1, 4, 7, and 9 compared with other clusters (Figure 4E). Notably, clusters 1 and 9 exhibited higher levels of chromatin accessibility than clusters 4 and 7. Considering that clusters 4 and 7 correspond to protonema and protonema apical stem cells, respectively, while clusters 1 and 9 correspond to reprogramming leaf cells and potentially incomplete reprogramming cells, these findings indicate that protonema cells exhibit a more relaxed chromatin structure compared with leaf cells. Furthermore, the data suggest that chromatin undergoes even greater relaxation during the transition from leaf cells to protonema apical stem cells, reflecting the chromatin remodeling required for cellular reprogramming. Together, these indicate that wounding creates a relaxed state of chromatin, leading to alterations in the transcriptional network.

### Changes in chromatin accessibility at STEMIN1 target genes

**Figure 5:**
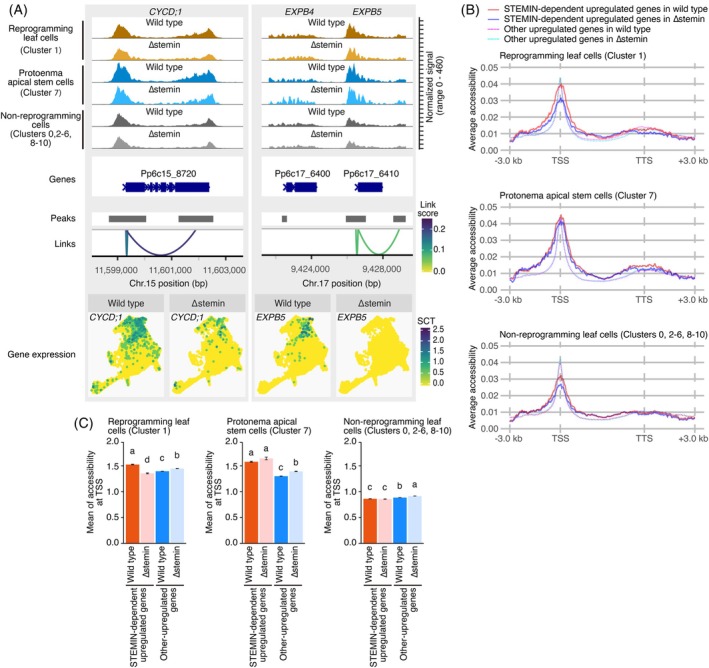
Correlation between gene expression and chromatin accessibility. (A) Distribution of ATAC‐seq peaks at the *CYCD;1* and *EXPB5* loci in reprogramming leaf cells (Cluster 1), protonema apical stem cells (cluster 7), and other cells (clusters 0, 2, 3, 4, 5, 6, 8, 9, and 10) from wild‐type and ∆stemin plants. Peaks are associated with gene expression, with link scores representing Pearson's correlation, color‐coded as shown. UMAP visualization of CYCD;1 and EXPB5 expression in wild‐type and ∆stemin plants is also shown. Note that the *CYCD;1* UMAP is identical to that in Figure 2F. (B) Distribution of ATAC‐seq peaks on STEMIN‐dependent upregulated 54 genes and each cluster‐specific upregulated genes in reprogramming leaf cells (cluster 1), protonema apical stem cells (cluster 7), and the others (clusters 0, 2, 3, 4, 5, 6, 8, 9, and 10) of wild‐type ∆stemin plants. (C) Mean of chromatin accessibility at around TSS (2 kb upstream of the TSS to 200 bp downstream of the TSS) in 54 STEMIN‐dependent and other upregulated genes of wild‐type and ∆stemin plants. Lowercase letters indicate significant differences (one‐way ANOVA and Tukey HSD test, *P* < 0.05). Error bars exhibit SE.

To examine the chromatin landscape of STEMIN1‐regulated genes, we analyzed chromatin accessibility at the *CYCD;1* and *EXPB5* loci across different cell clusters: reprogramming leaf cells (cluster 1), protonema apical stem cells (cluster 7), and other non‐reprogramming cells (clusters 0, 2, 3, 4, 5, 6, 8, 9, and 10: Figure 5A). This analysis was performed separately for wild‐type and ∆stemin plants. At the *CYCD;1* locus, chromatin accessibility peaks were detected at the transcription start site (TSS) and transcription termination site (TTS) across all cell clusters. Notably, protonema apical stem cells exhibited increased chromatin accessibility at the TSS and within the region spanning exon 6 to the TTS, with this enrichment being more pronounced compared with non‐reprogramming cells in both genotypes. In reprogramming cells, the ATAC‐seq peaks displayed an intermediate level of chromatin accessibility to those of stem cells and differentiated cells. However, in both reprogramming leaf cells and protonema apical stem cells, these peaks were more prominent in wild‐type plants than in ∆stemin plants. Similarly, *EXPB5* exhibited a chromatin accessibility pattern comparable to *CYCD;1*, displaying STEMIN‐dependent peak changes in both the reprogramming and protonema stem cell clusters (Figure 5A). Additionally, expression of *CYCD;1* and *EXPB5* was decreased or repressed in the ∆stemin plants (Figure 5A). These findings indicate that STEMIN enhances chromatin accessibility at these loci during the reprogramming of gametophore leaf cells into protonema apical stem cells.

We next analyzed average peaks of 54 genes specifically upregulated in a STEMIN‐dependent manner (referred to hereafter as “54 STEMIN‐dependent genes”), including *CYCD;1* and *EXPB5*, in wild‐type and ∆stemin plants. These 54 genes were compared with the remaining genes specifically upregulated in reprogramming cell cluster (referred to hereafter as “other upregulated genes”). Chromatin accessibility was assessed by calculating the average fragment count within 100 bp increments across a 3‐kb window surrounding each gene (1.5 kb upstream of the TSS and 1.5 kb downstream of the TTS) (Figure 5B). We also calculated the mean of total fragment count from 2 kb upstream of the TSS to 200 bp downstream of the start codon for both the 54‐ and other upregulated genes in wild‐type and ∆stemin plants (Figure 5C).

In wild‐type plants, the chromatin accessibility peaks around the TSS of the 54 STEMIN‐dependent genes, and other upregulated genes were comparatively low in non‐reprogramming cells but elevated in reprogramming leaf cells and protonema apical stem cells (Figure 5B,C). However, in ∆stemin plants, these chromatin accessibility peaks in protonema apical stem cells and non‐reprogramming cells were not significantly different from those in wild‐type plants, whereas the peak in reprogramming leaf cells was significantly reduced (Figure 5B, C). In contrast, for the other upregulated genes, the chromatin accessibility peaks tended to be higher in ∆stemin plants across all cell types. These findings suggest that STEMINs enhance chromatin accessibility specifically at the TSS of genes crucial for stem cell formation within a broadly permissive chromatin environment. This localized increase in accessibility likely facilitates the transcriptional activation of key regulatory genes during reprogramming. The attenuated effect in protonema apical stem cells implies that STEMIN's role in modulating chromatin accessibility is context‐dependent and may be influenced by additional regulatory factors across different cell types or developmental stages.

### Potential transcription factors involved in reprogramming cells

**Figure 6:**
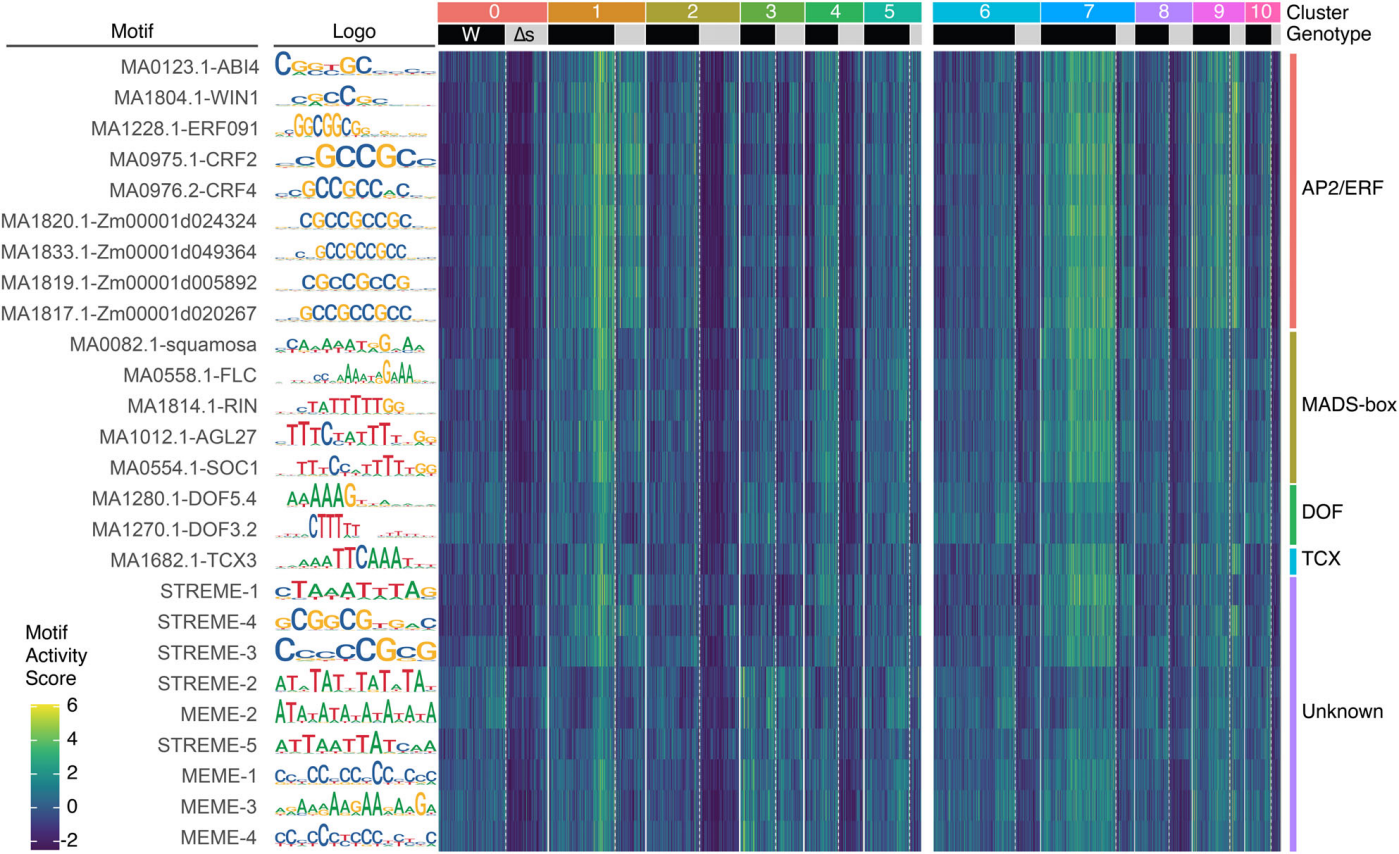
Potential transcription factors involved in reprogramming cells. Prediction of transcription factor binding sites significantly enriched in the promoter regions of genes upregulated in the reprogramming cell cluster (cluster 1) of combined wild‐type and ∆stemin lines using MEME (multiple expression motifs for motif elicitation) motif discovery tool. The names of the known transcription factors and their corresponding motif logos are shown on the left. The heatmap reveals the enrichment levels of motifs significantly enriched in cluster 1 across other clusters.

Next, to identify potential transcription factors functioning in reprogramming, we performed peak calling on the chromatin accessibility data using MACS2 (Zhang *et al*., 2008). Subsequently, we conducted a Wilcoxon rank sum test comparing differential gene expression between cells of the reprogramming cluster and other all other cells, and indentified peaks of accessibility that correlated with these genes. We then focused on peaks located within the 2‐kb promoter region upstream of the TSS. Motif analysis using the XTREME algorithm in MEME Suite version 5.5.5 (Bailey et al., 2015; Grant & Bailey, 2021) was performed on these promoter‐associated peaks. For background peaks, we used the MatchRegionStats function of the Signac package (Stuart et al., 2021) to return an equal number of peaks with a similar GC content as our differentially accessible peaks. This motif analysis identified 26 binding sites for transcription factors (Figure 6). Specifically, we found the enrichment of potential motifs for AP2/ERF‐type transcription factors, ERF091, CYTOKININ RESPONSE FACTOR 2 (CRF2) and CRF4 (Rashotte et al., 2006), ABA‐INSENSITIVE 4 (ABI4; Finkelstein et al., 1998), and WAX INDUCER 1 (WIN1; Broun et al., 2004) in reprogramming cells of both wild‐type and ∆stemin plants. Additionally, five motifs (MA1820.1, MA1833.1, MA1819.1, MA1817.1, and MA0975) exhibited similarity to the GCC‐box, a *cis*‐element recognized by AP2/ERF‐type transcription factors (Allen et al., 1998; Ohme‐Takagi & Shinshi, 1995) (Figure 6). While these 10 AP2/ERF‐related motifs were enriched in reprogramming cells (cluster 1) and potentially incomplete reprogramming cells (cluster 9) in both genotypes, their enrichment was observed exclusively in wild‐type protonema apical stem cells (cluster 7). These findings suggest that wounding stimuli induce a STEMIN‐independent relaxation of the chromatin regions of genes targeted by multiple AP2/ERF transcription factors, which initiates the AP2/ERF‐mediated transcriptional regulatory network. Subsequently, the function of STEMIN may contribute to the maintenance of this state to become protonema apical stem cells.

In addition to AP2/ERF motifs, we identified potential motifs for members of the MADS‐box family, SQUAMOSA (Huijser et al., 1992), FLOWERING LOCUS C (FLC; Hepworth, 2002), RIPENING INHIBITOR (RIN; Vrebalov et al., 2002), AGAMOUS‐LIKE 27 (AGL27; Alvarez‐Buylla et al., 2000), and SUPPRESSOR OF OVEREXPRESSION OF CO 1 (SOC1; Samach et al., 2000), which were enriched in not only reprogramming leaf cells but also protonema apical stem cells, depending on *STEMIN* genes (Figure 6). Motifs for DNA‐BINDING ONE FINGER (DOF) transcription factor 3.2 (DOF3.2) and DOF5.4 (Yanagisawa, 2002), TESMIN/TSO1‐LIKE CXC DOMAIN‐CONTAINING PROTEIN 3 (TCX3)/SOL1 (Andersen et al., 2007; Hauser et al., 2000) and some unidentified transcription factors were also enriched in reprogramming cells in a STEMIN‐dependent manner. These indicate a requirement for STEMIN activity in their chromatin relaxation in reprogramming cells.

Together, these motif analyses suggest that wounding initiates chromatin relaxation for the activation of AP2/ERF target genes and STEMIN activity subsequently enhances chromatin accessibility at the loci of MADS‐box, DOF, and TCX transcription factor targets, facilitating further reprogramming events. This might highlight a multi‐step process regulating chromatin structural changes during cellular reprogramming.

## DISCUSSION

Precise control of gene expression is fundamental for establishing and maintaining distinct cellular identities. In eukaryotic cells, chromatin accessibility, which dictates the availability of DNA for transcription, profoundly influences transcriptional outcomes. In this study, we investigated the interplay between gene expression and chromatin dynamics during STEMIN‐mediated reprogramming in *P. patens* following wounding and found a hierarchical chromatin remodeling process that accompanies the transition from differentiated leaf cells to stem cells. In addition, we found that the AP2/ERF transcription factor STEMIN selectively enhances accessibility at specific genomic loci essential for stem cell formation within this relaxed chromatin state. These results suggest a hierarchical chromatin remodeling during the reprogramming process. Initially, wounding acts as a trigger to induce a permissive chromatin state through broad changes in chromatin accessibility, potentially facilitated by certain AP2/ERF transcription factors (Figure 6). This suggests the initiation of gene regulatory networks governed by multiple AP2/ERF transcription factors. This primed cellular environment is then refined by STEMIN, which selectively targets specific chromatin regions to activate genes required for stem cell formation.

Several AP2/ERF transcription factors have been implicated in wound‐induced reprogramming across diverse plant lineages. For instance, LOW‐AUXIN RESPONSIVE in *Marchantia polymorpha* (Ishida et al., 2022), and WIND and ERF115 in *A. thaliana*, play crucial roles in cell fate transitions, similar to STEMIN in *P. patens*. While the precise mechanisms by which these factors influence chromatin accessibility remain to be fully elucidated, the observation that these factors alone can induce cellular state transitions suggests that they may share a conserved capacity to regulate chromatin states. This regulation likely occurs in response to wounding and potentially other environmental cues, although lineage‐specific variations in the specific target genes and downstream effects are expected. The ability of these transcription factors to modulate chromatin accessibility may have conferred a significant evolutionary advantage, enabling plants to rapidly and efficiently adjust to fluctuating environmental conditions while simultaneously maintaining robust developmental control.

In both mammals and land plants, specific transcription factors can reprogram one cell type into another by inducing changes in chromatin structures (Barral & Zaret, 2024; Lai et al., 2018). In mammals, stem cells do not naturally regenerate after embryogenesis, yet the induction of four transcription factors (Oct4, Sox2, Nanog, and c‐Myc) can reprogram chromatin states and convert somatic cells into induced pluripotent stem (iPS) cells (Takahashi & Yamanaka, 2006). While this direct cell reprogramming appears to be a stochastic process that depends on the amount, balance, continuity, and silencing of expression of the factors (Buganim et al., 2012; Hanna et al., 2009), Oct4 and Sox2 act as pioneer factors that are dominant in their ability to engage silent chromatin and activate the expression of their target genes with other transcription factors, thereby initiating new gene regulatory networks (Soufi et al., 2012). Similarly, in *A. thaliana*, LEAFY encoding a helix‐turn‐helix transcription factor has been characterized as a pioneer transcription factor, initiating floral development by directly binding to closed chromatin regions of the floral commitment gene *APETALA1* (Jin et al., 2021; Lai et al., 2021).

Unlike these pioneer transcription factors, STEMIN1 does not appear to directly convert closed chromatin into an open state, although ectopic expression of STEMIN1 is able to induce cell fate changes. Instead, it preferentially binds to annotated TSS (Ishikawa et al., 2019) that exhibit pre‐existing open chromatin regions (Figure 6). Rather than acting as a pioneer factor, STEMIN seems to refine chromatin accessibility at TSSs within a permissive chromatin environment. Additionally, ectopic induction of STEMIN1 in leaf cells leads to a reduction in histone H3 lysine 27 trimethylation (H3K27me3), a repressive chromatin modification, on the coding regions of its direct target genes before cell division (Ishikawa et al., 2019). While the precise relationship between this H3K27me3 reduction on coding sequences and the enhancement of chromatin accessibility near TSSs remains to be fully elucidated, this hierarchical model of chromatin opening underscores the precise regulation of gene expression required for stem cell formation through multi‐step control. Whether STEMIN‐mediated TSS refinement and local H3K27me3 reduction on the coding regions influence each other or are regulated independently requires further investigation.

Additionally, we found a STEMIN‐dependent upregulation of *ATAXIA‐TELANGIECTASIA MUTATED AND RAD3‐RELATED* (*ATR*), a key mediator of single‐strand DNA break responses in both animals and plants (Yoshiyama et al., 2013) (Figure S9). Furthermore, we detected upregulation of *SUPPRESSOR OF GAMMA RESPONSE 1A* (*SOG1A*), a *P. patens* homologue of the DNA damage response gene *SOG1* in *A. thaliana* (Sakamoto et al., 2021), alongside its potential downstream targets, including *WEE1A*, *RAD51B*, *SMR9*, and *SMR10*, in reprogramming cells (Figure S11). Notably, transient DNA damage induced by DNA strand‐breaking chemicals can reprogram leaf cells into stem cells through ATR activation and subsequent expression of *STEMIN* genes (Gu et al., 2020). These suggest a potential link between the DNA damage response pathway and STEMIN‐mediated reprogramming triggered by wounding.

In mice, single‐strand DNA breaks occur during germ cell formation, concurrently with base excision repair activation. This process is mechanistically linked to genome‐wide DNA demethylation, leading to large‐scale chromatin remodeling (Hajkova et al., 2010). While the DNA damage response pathway is primarily designed to maintain genome stability, its integration into reprogramming mechanisms could enable precise regulation of chromatin states and gene expression during cell fate transitions. Whether DNA damage occurs naturally during reprogramming in *P. patens* remains unknown, but our findings suggest a potential feedback loop between STEMIN activity and the DNA damage response pathway. This interplay may contribute not only to genome‐wide chromatin relaxation but also to the activation of target genes through selective chromatin remodeling mediated by STEMIN.

Beyond chromatin dynamics, autophagy has been increasingly recognized as a key regulator of cellular reprogramming (Petersen et al., 2024). Overexpression of *AUTOPHAGY 8* (*ATG8*), also known as *MICROTUBULE‐ASSOCIATED PROTEIN 1 LIGHT CHAIN 3* (*LC3*), in gametophores enhances the reprogramming capacity of leaf cells (Kanne et al., 2022). Conversely, the subsequent downregulation of autophagic activity in reprogrammed chloronema apical stem cells suggests a temporally restricted role for autophagy, likely being activated prior to or during the early stages of reprogramming (Kanne et al., 2022). This activation may facilitate the degradation of cellular components associated with the preceding differentiated state, thereby promoting a smoother transition to a new cell fate. The upregulation of autophagy‐related genes in reprogramming leaf cells of the ∆stemin plant (Figure S7) suggests that STEMINs function to suppress autophagy during the transition from differentiated cells to stem cells.

In *A. thaliana*, autophagy has also been implicated in callus formation (Rodriguez et al., 2020) and graft‐induced wound healing (Kurotani et al., 2025), underscoring its broader role in regeneration and cellular reprogramming across land plants. Future studies should aim to elucidate the molecular mechanisms governing the interplay between AP2/ERF transcription factors, such as STEMIN in *P. patens* and WIND in *A. thaliana*, and the autophagy machinery, with particular emphasis on how this interaction influences chromatin accessibility and regulates cell fate transitions in response to developmental and environmental cues throughout land plant lineages.

## MATERIALS AND METHODS

### Plant materials and growth conditions

The Gransden 2004 strain of *Physcomitrium patens* (Hedw.) Mitt. (Rensing et al., 2008) was used as wild‐type in this study. ∆stemin1∆stemin2∆stemin3#6–48‐1 (∆stemin) plant (Gu et al., 2020) was used as a triple‐deletion mutant of *STEMIN*s. Wild‐type and ∆stemin plants were grown on solid BCDAT medium at 25°C under continuous white light for protonema propagation. For the induction of gametophores, protonemata were propagated on solid BCDAT medium and cultured at 25°C under continuous white light for 3 weeks (Nishiyama et al., 2000). To induce reprogramming triggered by wounding, gametophores were collected into liquid BCDAT medium and chopped with a Polytron 1200E homogenizer (Ishikawa et al., 2011). Cut gametophores were washed with liquid BCDAT medium twice and then cultivated in liquid BCDAT medium.

### Single nuclei isolation and library preparation

Nuclei for the gametophore samples were isolated directly from tissue harvested from BCDAT medium‐grown plates after 22 days of cultivation. For time‐course experiments, 22‐day‐old tissue was subjected to mechanical disruption using a Polytron 1200E homogenizer. Nuclei were subsequently isolated at 3, 4.5, and 6 h (3–6 h timepoint), 10, 12, and 14 h (10–14 h timepoint), and 24 and 36 h (24–36 h time point) post‐disruption. To capture the endpoint of the reprogramming process, the 24–36 h timepoint samples were pooled with protonemata 1 week after disruption with a homogenizer (24–36 h + protonemata), ensuring sufficient representation of differentiated protonema cells for subsequent analyses.

Nuclei were isolated by chopping collected tissue in 3 mL of chopping buffer (1% [w/v] BSA; 10 mM NaCl; 10 mM Tris–HCl, pH 7.5; 3 mM MgCl_2_; 1 mM DTT; 0.5 mM spermine; 0.5 mM spermidine; 1 U/μL Protector RNase Inhibitor, 1× cOmplete Mini, EDTA‐free Protease Inhibitor Cocktail [Roche], 1% [w/v] PVP40; 0.3% [v/v] NP‐40; 0.1% [v/v] Tween 20 in RNase free water) for 3 min using a double‐edged safety razor (FEATHER) in a 5 mL culture dish on ice. After a 2‐min incubation, the tissue was strained through a 20 μm CellTrics filter (Sysmex). The filter was rinsed with 1 mL of rinsing buffer (1% [w/v] BSA; 10 mM NaCl; 10 mM Tris–HCl, pH 7.5; 3 mM MgCl_2_; 1 mM DTT; 0.5 mM spermine; 0.5 mM spermidine; 1 U/μL Protector RNase Inhibitor, 1 × cOmplete Mini, EDTA‐free Protease Inhibitor Cocktail [Roche], 0.5 μg/mL 4′,6‐diamidino‐2‐phenylindole [DAPI]). The DAPI stained nuclei were isolated by fluorescence‐activated nuclei sorting (FANS) using a BD FACS Melody fitted with a 100 μm sorting nozzle. Nuclei were sorted into a collection tube filled with 500 μL of rinsing buffer kept at 4°C. To ensure equal representation across subsamples, nuclei were pooled during sorting, collecting 20 000 nuclei for each of three subsamples, resulting in a total of 60 000 nuclei per sample. Collected nuclei were centrifuged at 500*g* for 3 min with the centrifuge brake off in a swing‐bucket centrifuge. Excess buffer was carefully removed, leaving a pellet of nuclei in approximately 10 μL of buffer. Nuclei were gently mixed in the remaining buffer by pipetting with a wide bore tip.

For quality control, 1 μL of the prepared nuclei suspension was diluted in 9 μL of rinsing buffer and counted using a hemocytometer (Neubauer). Five microliters of isolated nuclei were used to generate single‐cell libraries according to the user guide (CG000338 Rev E) for the Chromium Next GEM Single Cell Multiome ATAC + Gene Expression kit (10xGENOMICS).

### Library sequencing

For snRNA‐seq, libraries from the gametophores, 3–6 h and 10–14 h time points of wild‐type and ∆stemin plants were sequenced on the DNBSEQ (BGI), and the 24–36 h + protonemata libraries were sequenced on the NovaSeq 6000 (Illumina). For snATAC‐seq, libraries from the same samples were sequenced on the DNBSEQ (gameotophores, 3–6 h and 10–14 h) and NovaSeq 6000 (24–36 h + protonemata). All libraries were sequenced in dual‐index mode. For snRNA‐seq, index reads were sequenced with 10 cycles for both i7 and i5 indices, and paired‐end reads with 28 cycles for R1 and 90 cycles for R2. For snATAC‐seq, index reads were sequenced with 8 cycles for i7 and 24 cycles for i5, and paired‐end reads with 50 cycles for both R1 and R2.

### Data analysis

Sequence alignment and pre‐processing was performed using Cellranger‐ARC (10xGENOMICS). Version 6 of *Physcomitrium patens* genome (Bi et al., 2024: https://phytozome‐next.jgi.doe.gov) was used for alignment. Cellranger‐ARC output was analyzed using Seurat and Signac packages (Hao et al., 2024; Stuart et al., 2021).

For details on the packages used and the specific parameters selected for each function, the code is available at https://github.com/rdv‐nibb/P.patens_reprogramming_snMultiome. A brief summary of the functions and implementations is provided below. Count matrixes from Cellranger‐ARC were imported into *R* (version 4.4.2) and converted to gene expression and ATAC assays for eight multimodal Seurat objects, representing each time pooling and genotype, using the Seurat and Signac packages (Hao et al., 2024; Stuart et al., 2021). Following transcription start site (TSS) enrichment, cells with more than 5000 mapped snATAC‐seq reads, with TSS enrichment scores greater than 1, and with between 1000 and 20 000 mapped snRNA‐seq reads were selected, yielding a total of 20 883 reconstructed cells. Peaks of accessibility were estimated using MACS2 followed by normalization and dimensionality reduction through the Signac latent semantic indexing (LSI) pipeline. For the expression (RNA) assays, we employed the SCTransform function to correct RNA counts on a per sample basis followed by principal component analysis. Batch effect removal for both assays was performed using Harmony (Korsunsky et al., 2019). A multimodal, or weighted nearest‐neighbor graph was calculated from the two integrated assays, using the first 11 reductions from PCA, and the 2nd to the 13th reductions of the LSI. The neighbor graph was used for cluster estimation using a smart local moving algorithm (Waltman & Van Eck, 2013).

A custom reconstruction of the Version 6 genome of *P. patens* was generated through the BSGenome package (Pagès, 2024), allowing us to subsequently discern links between gene expression and peaks of accessibility and perform motif analysis.

For motif analysis, we first considered genes that were upregulated in the reprogramming cluster. Next, we considered regions annotated as peaks that correlated with the expression of these upregulated genes using the peak linkages of the Signac package. We collated sequences of these peak regions, if they overlapped with the promoter sequences (up to 2000 bp upstream of the TSS) of the associated genes, and submitted them for motif analysis to MEME Suite (version 5.5.7) using the XTREME motif discovery tool with default parameters, except as follows: for background peaks, we obtained an equal number of sequences that matched the region statistics of the peak sequences using the MatchRegionStats function of the Signac package, and for known motifs we selected the JASPAR Core 2022 collection for plants (Castro‐Mondragon et al., 2022).

To gauge the average accessibility for groups of genes in‐ or outside of the reprogramming context, we exported bigWig files for peak accessibility, with tile sizes of 100 bp for analysis with DeepTools version 3.5.5 (Ramírez et al., 2016). To obtain the mean accessibility for promoter regions (−2000 bp upstream of the TSS) plus the first 200 bp following the TSS, we generated a matrix of gene activity and compared genes annotated as upregulated during reprogramming to those regarded as upregulated during reprogramming in a STEMIN‐dependent manner. We did this comparison distinctly for each genotype and by specific subsets of cells (reprogramming leaf cells [cluster 1], protonema apical stem cells [cluster 7], and non‐reprogramming leaf cells [clusters 0, 2–6, 8–10]).

### Construction of CYCB;1‐GUS line and GUS staining

To insert the *uidA* gene (Jefferson, 1987) in frame with a *CYCB;1*‐coding sequence (Accession number: AB547330), a genomic DNA fragment of the gene extending from the middle to the last codon was PCR‐amplified from wild‐type genomic DNA. The amplified fragment was inserted into the 5'‐end of the coding region of the *uidA* gene in the pTN83 (accession: AB538275) plasmid in frame. Genomic fragments containing the 3'‐flanking region of the *CYCB;1* gene were inserted into the 3'‐region of the nptII expression cassette of the plasmids. The generated construct was digested by *Not*I and *Bam*HI restriction enzymes for gene targeting. Primers used for plasmid construction are as follows: 5'‐GGGGCGGCCGCAAAACCGAGGTCCAGAG‐3' and 5'‐GGGGGATCCAGCTGCTGGTTTGCGGGGAAC‐3' for 5' end of the CYCB;1‐coding region, and 5'‐GGGGTCGACCAAGCCGGCTACTGAATTCC‐3' and 5'‐GGGGGTACCTGAAGATACCCGATTGACGTC‐3' for the 3'‐flanking region of the *CYCB;1* gene.

GUS activity was detected as previously described (Nishiyama et al., 2000).

## Author Contributions

RV, GP, MH, and MI conceived and designed the research. RV, KA, and MI performed the experiments. RV, GP, KA, and YS analyzed the data. RV, MH, and MI wrote the manuscript and incorporated comments from all authors.

## Conflict of Interest

The authors declare that they have no competing interests.

## Supporting information


**Figure S1.** Quality control of scRNA‐seq and scATAC‐seq dataset after data filtering.
**Figure S2.** UMAP visualization of each cell cluster in combined wild‐type and ∆stemin samples.
**Figure S3.** Construction and characterization of CYCB;1‐GUS plants for visualization of gene expression patterns.
**Figure S4.** Dot‐plots of *PpVNS* and *PpHB7* genes in each cluster of combined wild‐type and ∆stemin samples.
**Figure S5.** Expression patterns of significantly enriched genes in each cluster of wild‐type and ∆stemin plants.
**Figure S6.** Expression patterns of DEGs identified in reprogramming leaf cells at 0 and 24 h post‐excision using 1cell‐DGE.
**Figure S7.** Dot‐plots of autophagy‐related genes in each cluster of wild‐type and ∆stemin plants.
**Figure S8.** Hypothetical model illustrating the functional relationship between *STEMIN* and *PpWOX13L* in reprogramming leaf cells.
**Figure S9.** Hypothetical model for STEMIN‐mediated gene expression in response to wounding.
**Figure S10.** Correlation between gene expression and chromatin accessibility of all genes expressed in each cluster.
**Figure S11.** Dot‐plots of genes involved in DNA damage response/repair in each cluster of wild‐type and ∆stemin plants.

**Table S1.** Quality control of snRNA‐seq and snATAC‐seq of each sample.
**Table S2.** List of genes differentially expressed specifically in reprogramming leaf cells (cluster 1).
**Table S3.** List of 54 STEMIN‐dependent upregulated genes and 108 STEMIN‐dependent downregulated genes in reprogramming leaf cells (cluster 1).

## Data Availability

All relevant data can be found within the manuscript and its supporting materials. The sequence data that support the findings of this study are openly available in National Center for Biotechnology Information at https://www.ncbi.nlm.nih.gov/geo/query/acc.cgi?acc=GSE293939, reference number GSE293939. Materials used in this study are available from the corresponding authors upon reasonable request. The code used in this study can be accessed at https://github.com/rdv‐nibb/P.patens_reprogramming_snMultiome.
