# Supplementary material for: STEMIN transcription factor drives selective chromatin remodeling for gene activation within a relaxed chromatin during reprogramming in the moss Physcomitrium patens
Source: Plant J. 2025 Aug 1;123(3):e70386. doi: 10.1111/tpj.70386 (PMC12316476; doi:10.1111/tpj.70386)
Supplement: Supplementary file 1 — Figure S1. Quality control of scRNA‐seq and scATAC‐seq dataset after data filtering. Figure S2. UMAP visualization of each cell cluster in combined wild‐type and ∆stemin samples. Figure S3. Construction and characterization of CYCB;1‐GUS plants for visualization of gene expression patterns. Figure S4. Dot‐plots of PpVNS and PpHB7 genes in each cluster of combined wild‐type and ∆stemin samples. Figure S5. Expression patterns of significantly enriched genes in each cluster of wild‐type and ∆stemin plants. Figure S6. Expression patterns of DEGs identified in reprogramming leaf cells at 0 and 24 h post‐excision using 1cell‐DGE. Figure S7. Dot‐plots of autophagy‐related genes in each cluster of wild‐type and ∆stemin plants. Figure S8. Hypothetical model illustrating the functional relationship between STEMIN and PpWOX13L in reprogramming leaf cells. Figure S9. Hypothetical model for STEMIN‐mediated gene expression in response to wounding. Figure S10. Correlation between gene expression and chromatin accessibility of all genes expressed in each cluster. Figure S11. Dot‐plots of genes involved in DNA damage response/repair in each cluster of wild‐type and ∆stemin plants. [file TPJ-123-0-s002.pdf]

SUPPORTING INFORMATION

STEMIN transcription factor drives selective chromatin remodelling for gene activation within a relaxed chromatin during reprogramming in the moss *Physcomitrium patens*

Ruan Morné de Villiers, Gergő Pálfalvi, Akinori Kanai, Yutaka Suzuki, Mitsuyasu Hasebe, and Masaki Ishikawa

SUPPORTING FIGURES

Figure S1. Quality control of scRNA-seq and scATAC-seq dataset after data filtering.
Figure S2. UMAP visualization of each cell cluster in combined wild-type and Δstemin samples.
Figure S3. Construction and characterization of CYCB;1-GUS plants for visualization of gene expression patterns.
Figure S4. Dot-plots of *PpVNS* and *PpHB7* genes in each cluster of combined wild-type and Δstemin samples.
Figure S5. Correlation between gene expression and chromatin accessibility of all genes expressed in each cluster.
Figure S6. Expression patterns of DEGs identified in reprogramming leaf cells at 0 and 24 h post-excision using 1cell-DGE.
Figure S7. Dot-plots of autophagy-related genes in each cluster of wild-type and Δstemin plants.
Figure S8. Hypothetical model illustrating the functional relationship between *STEMIN* and *PpWOX13L* in reprogramming leaf cells.
Figure S9. Hypothetical model for STEMIN-mediated gene expression in response to wounding.
Figure S10. Correlation between gene expression and chromatin accessibility of all genes expressed in each cluster.
Figure S11. Dot-plots of genes involved in DNA damage response/repair in each cluster of wild-type and Δstemin plants.

SUPPORTING TABLES

Table S1. Quality control of snRNA-seq and snATAC-seq of each sample.
Table S2. List of genes differentially expressed specifically in reprogramming leaf cells (cluster 1).
Table S3. List of 54 STEMIN-dependent upregulated genes and 108 STEMIN-dependent downregulated genes in reprogramming leaf cells (cluster 1).

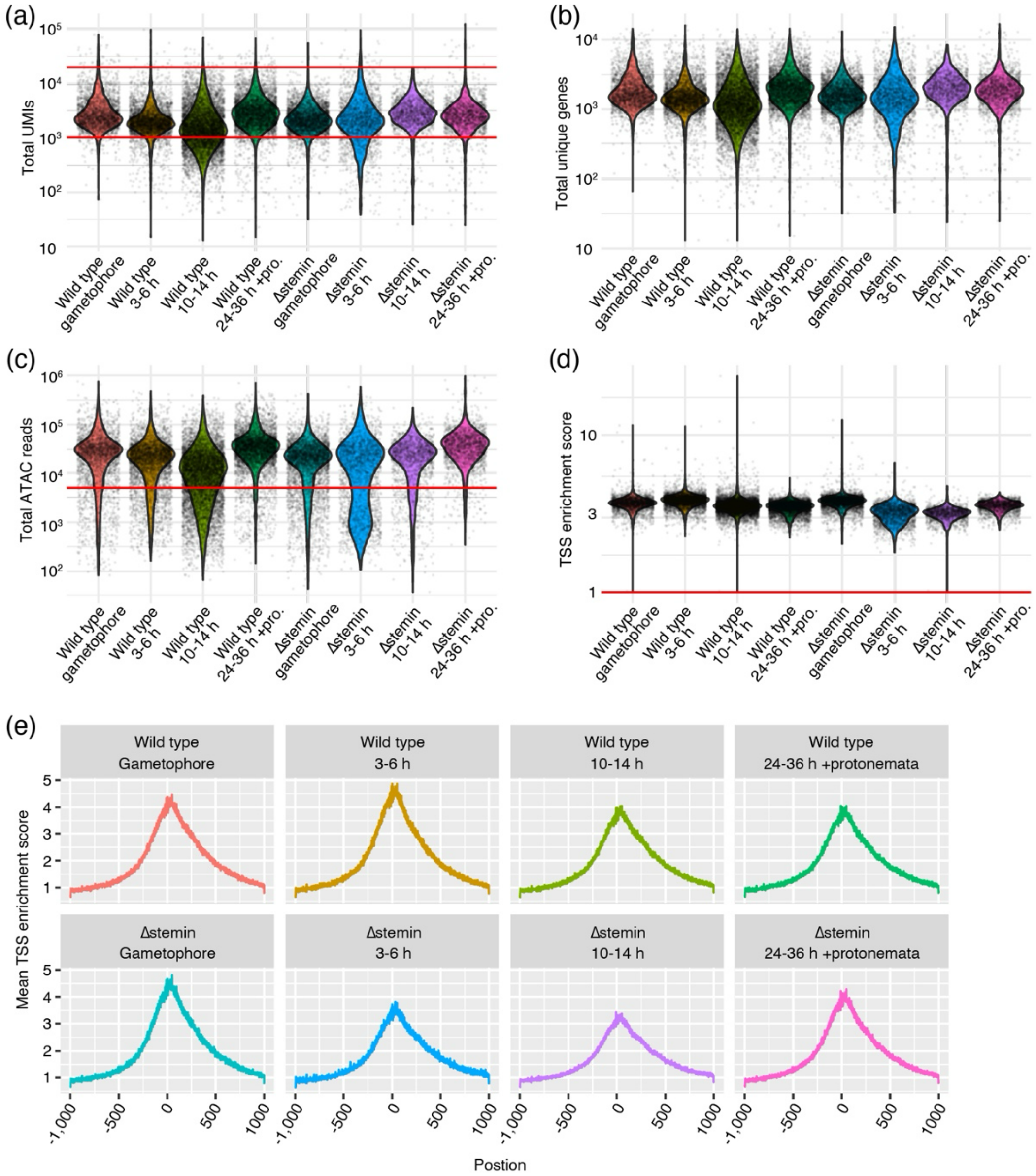

Figure S1. Quality control of snRNA-seq and snATAC-seq dataset after data filtering.

(a) Distribution of the number of UMIs per cell in each sample. Red lines indicate the minimal (1,000) and maximal (20,000) UMI thresholds per cell. See the text for details.

(b) Distribution of the number of genes per cell in each sample.

(c) Distribution of the number of ATAC-seq reads in each sample. The red line indicates the minimal ATAC read threshold (5,000) per cell.

(d) Transcriptional starting site (TSS) enrichment score of each sample. The red line indicates the minimal TSS enrichment score threshold (1) per cell.

(e) The mean of TSS enrichment score of each sample.

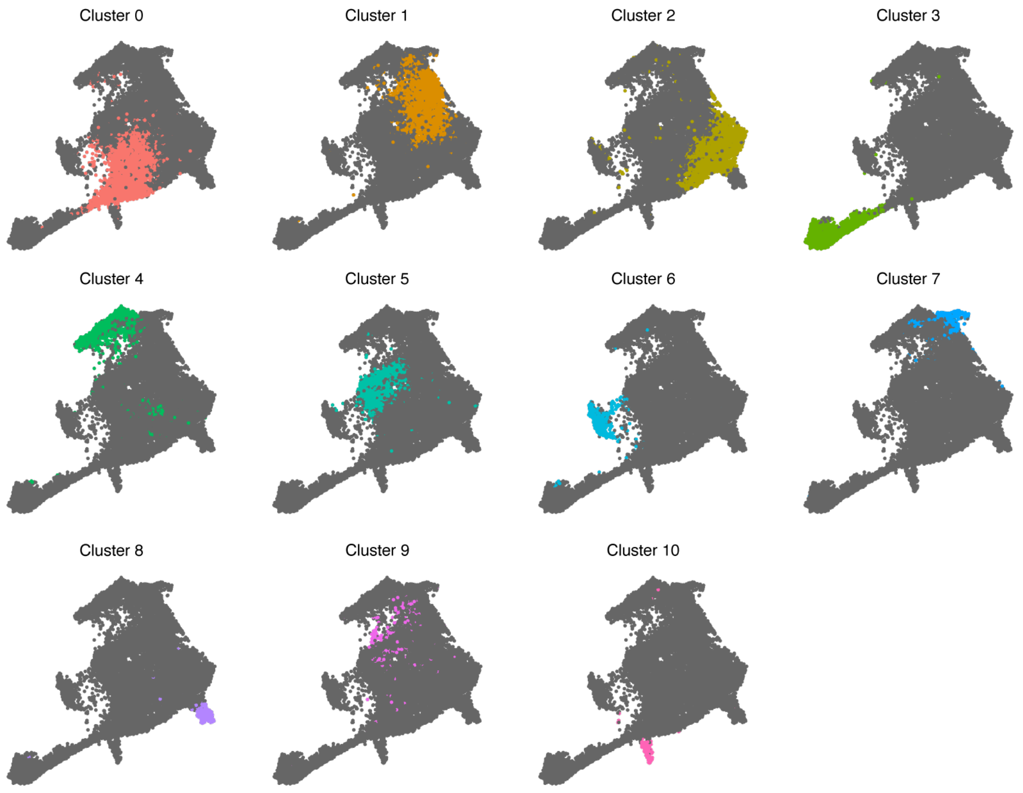

Figure S2. UMAP visualization of each cell cluster in combined wild-type and Δstemin samples
Cells within cluster 0 to 10 are shown in different colours on UMAP.

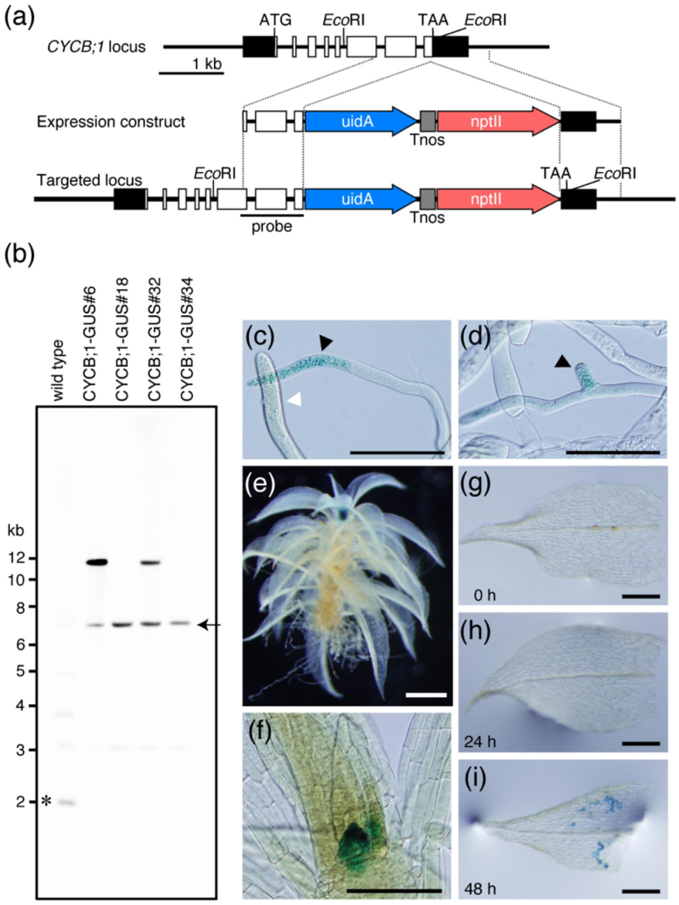

Figure S3. Construction and characterization of CYCB;1-GUS plants for visualization of gene expression patterns.

(a) Schematic diagrams of the targeting strategy for the *CYCB;1* locus. White and black boxes represent coding sequences and untranslated regions, respectively. Blue and light red arrow boxes denote the *uidA* gene (GUS; Jefferson *et al.*, 1987) and the neomycin phosphotransferase II expression cassette (nptII; Nishiyama *et al.*, 2000), respectively. Probes used in (b) are shown.

(b) DNA gel blot analysis of candidate targeted lines. Genomic DNA of wild-type and CYCB;1-GUS (#6, #8, #32 and #34) plants was digested with *Eco*RI. The asterisk and arrow indicate DNA fragments specific to wild-type and CYCB;1-GUS plants, respectively, using the specific primer shown in (a).

(c-i) Representative GUS expression pattern of protonemata (c,d), gametophore (e), a shoot apex of a gametophore (f), and cut leaves (0 h [g], 24 h [h], and 48 h [i]) in the CYCB;1-GUS#18 line. Black and white arrowheads denote the GUS-positive and -negative protonema apical stem cells in protomenata, respectively. Scale bars: 100 µm in (c,d); 500 µm in (e); 100 µm in (f); and 200 µm in (g-i).

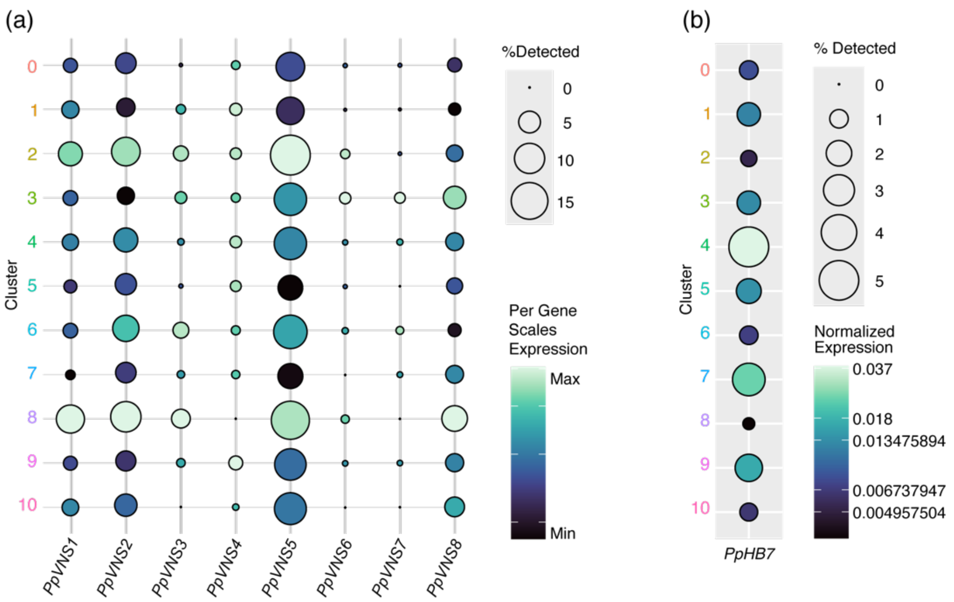

Figure S4. Dot-plots of *PpVNS* and *PpHB7* genes in each cluster of combined wild-type and Δstemin samples.

Dot diameters and colours denote the proportion of cells and the average expression levels of the *PpVNS1* to *PpVNS8* (a) and *PpHB7* (b), respectively.

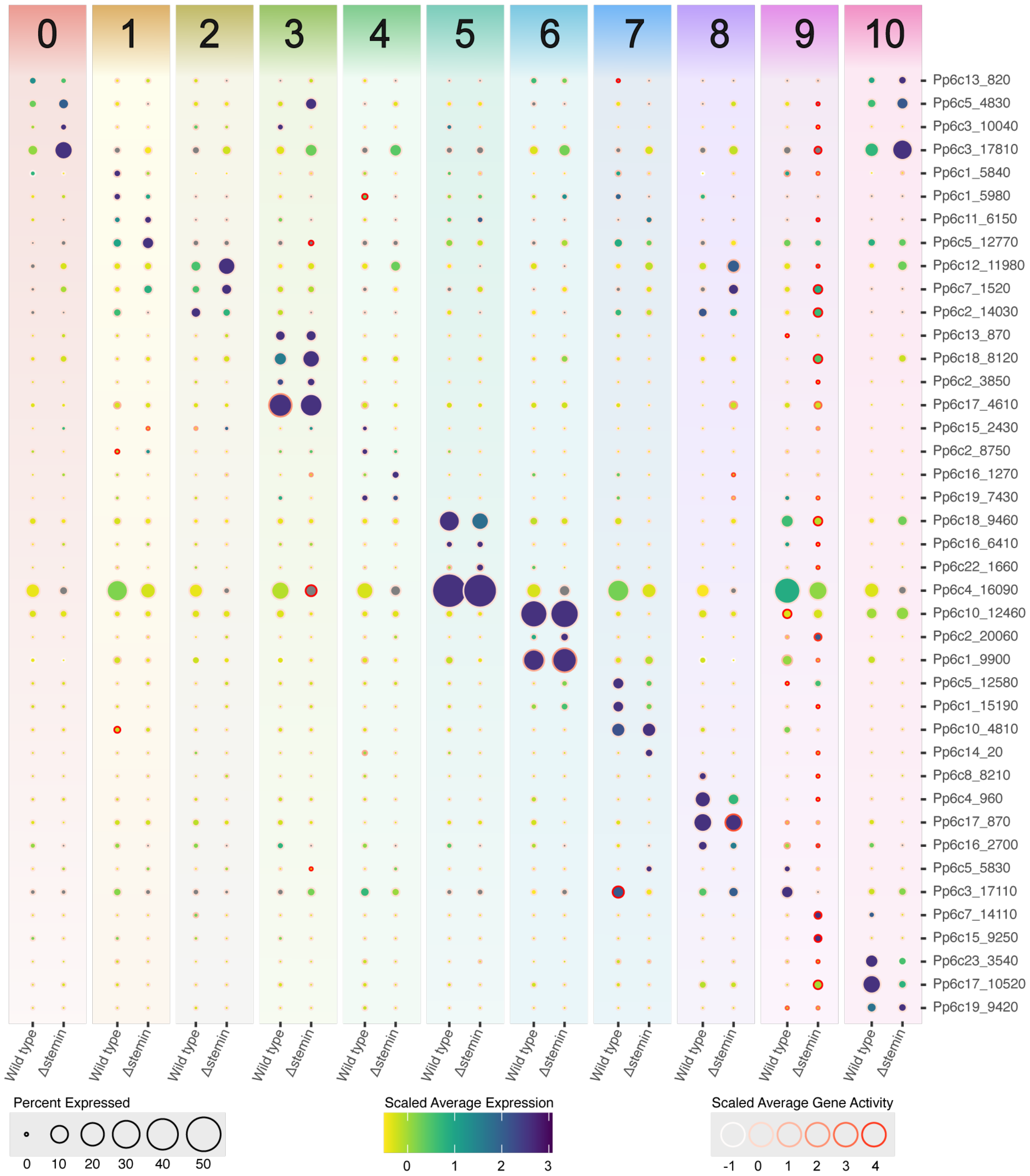

Figure S5. Expression patterns of significantly enriched genes in each cluster of wild-type and Δstemin plants.

Dot diameter indicates the proportion of cluster cells expressing a given gene. The colour bar denotes the relative expression level. Dot outline indicates the relative chromatin accessibility for the gene.

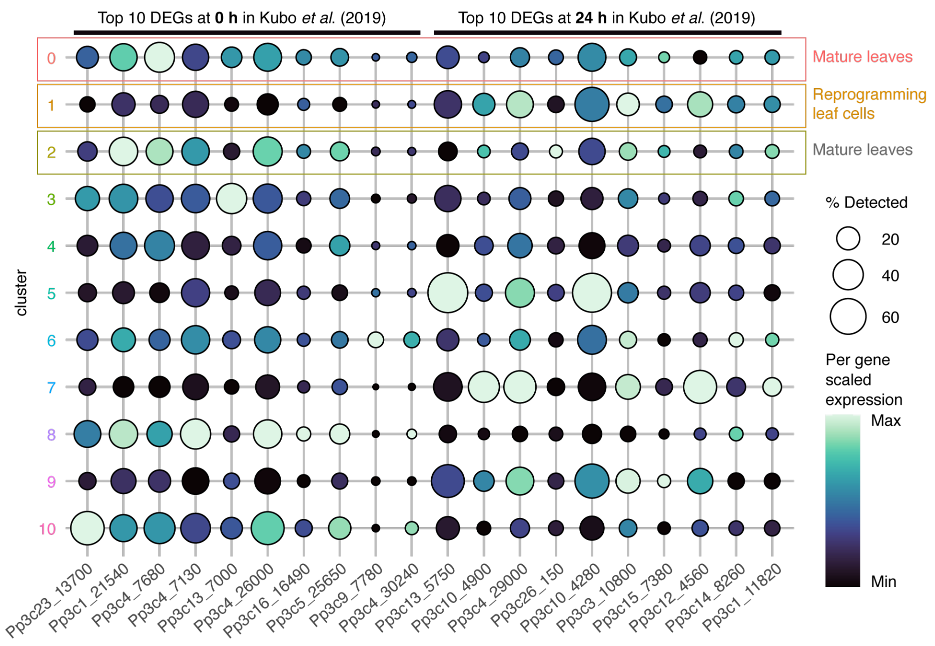

Figure S6. Expression patterns of DEGs identified in reprogramming leaf cells at 0 and 24 h post-excision using 1cell-DGE.

Dot diameter indicates the proportion of cluster cells within each cluster that express the corresponding gene. The colour bar denotes the relative expression level. The top 10 DEGs at 0 h identified by 1cell-DGE (Kubo *et al*., 2019): *Pp3c23_13700* (unknown), *Pp3c1_21540* (aluminium induced protein-like), *Pp3c4_7680* (membrane protein, putative), *Pp3c4_7130* (unknown), *Pp3c13_7000* (glyoxal oxidase-related protein-like), *Pp3c4_26000* (chaperone DnaJ-domain superfamily protein-like), *Pp3c16_16490* (unknown), *Pp3c5_25650* (unknown), *Pp3c9 _7780* (calcium-dependent lipid-binding amily protein-like), and *Pp3c4_30240* (TOXICOS ENLEVADURA 2-like). The top 10 DEGs at 24 h (Kubo *et al*., 2019): *Pp3c13_5750* (lactoyl-glutathione lyase/glyoxalase I family protein-like), *Pp3c10_4900* (unknown), *Pp3c4_29000* (unknown), *Pp3c26_150* (unknown), *Pp3c10_4280* (bHLH protein), *Pp3c3_10800* (adenosine kinase 2-like), *Pp3c15_7380* (di-hydrodipicolinate reductase-like), *Pp3c12_4560* (expansinA9-like), *Pp3c14 _8260* (succinyl-CoA ligase, alpha subunit-like), and *Pp3c1_11820* (unknown).

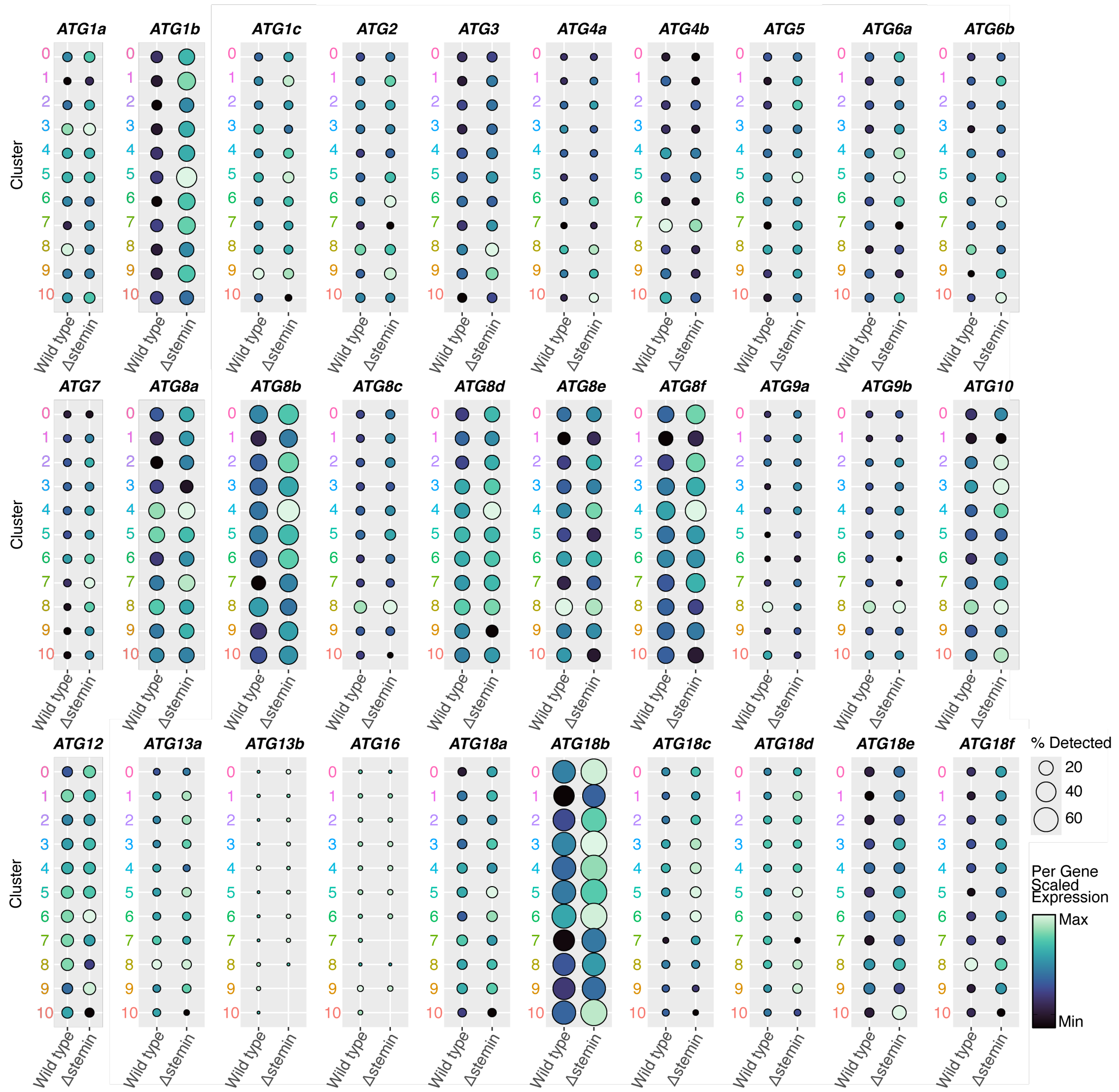

Figure S7. Dot-plots of autophagy-related genes in each cluster of wild-type and Δstemin plants.

Dot diameters and colours denote the proportion of cells and the average expression levels of the autophagy-related genes, respectively.

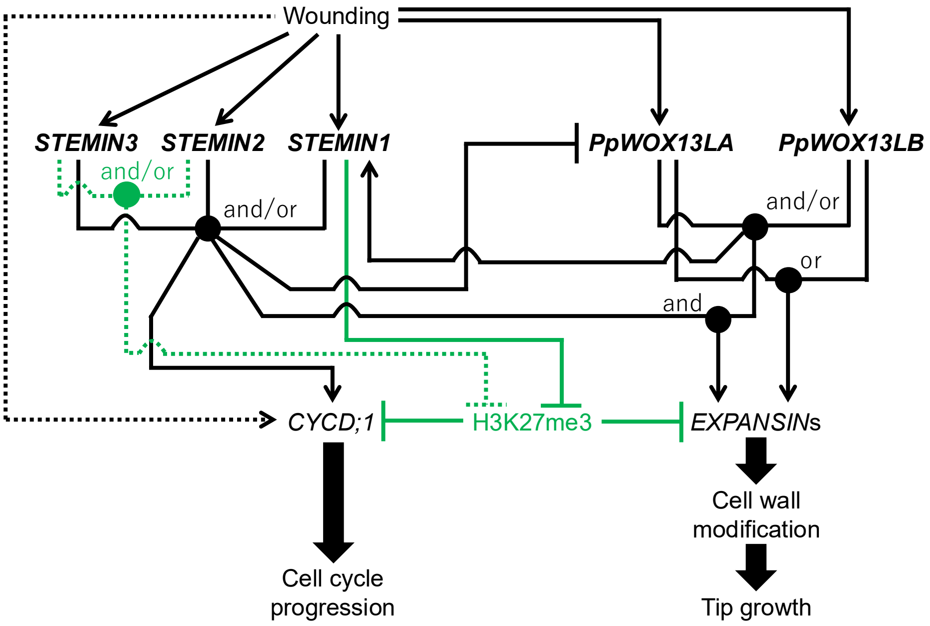

Figure S8. Hypothetical model illustrating the functional relationship between *STEMIN* and *PpWOX13L* in reprogramming leaf cells.

Wounding independently induces the expression of the three *STEMIN* and two *PpWOX13L* genes. *PpWOX13LA* and/or *PpWOX13LB* positively regulate *STEMIN1* expression, while *STEMIN1, STEMIN2*, and/or *STEMIN3* negatively regulate *PpWOX13LA* expression, but not *PpWOX13LB* (Fig. 2j,m). While *PpWOX13LA* and *PpWOX13LB* are indispensable for tip growth, the three *STEMIN* genes are not strictly required for this process. However, *STEMINs* regulate cell wall-modifying genes, such as *EXPANSINs* (Fig. 3c). This upregulation may be mediated through the reduction of H3K27me3 levels at these loci (shown by solid and dotted green lines; Ishikawa *et al.*, 2019) and/or through the integration of the *STEMIN*- and *PpWOX13L*-regulatory pathways. Additionally, *STEMIN*s promote cell cycle reactivation and progression by inducing the expression of *CYCD;1*, a function not attributed to *PpWOX13L* genes (Sakakibara *et al.*, 2014). Notably, *CYCD;1* is also activated via a *STEMIN*-independent mechanism, as its expression remains inducible by wounding even in lines lacking all three *STEMIN* genes (Fig. 2f,m). This suggests the existence of an alternative regulatory pathway, which is indicated by a dotted black arrow in the model.

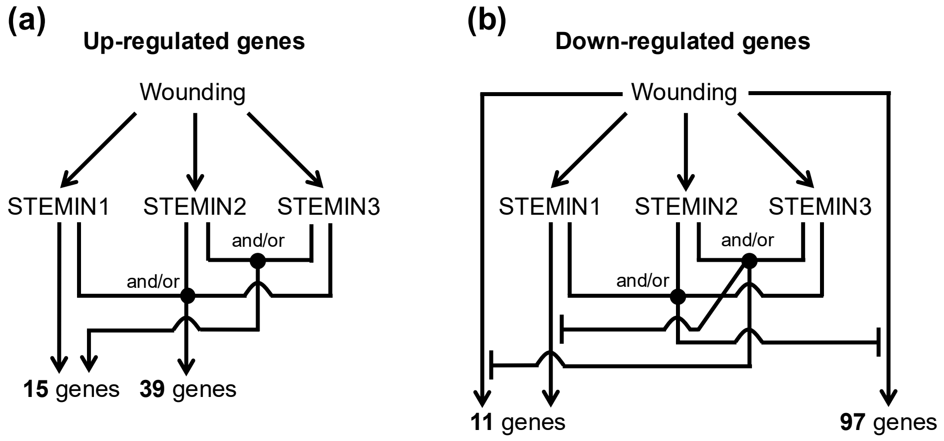

Figure S9. Hypothetical model for STEMIN-mediated gene expression in response to wounding.

(a) Upon wounding, 54 genes are upregulated in reprogramming leaf cells in a STEMIN-dependent manner (Fig. 3). Of these genes, 15 genes have been previously annotated as direct targets of STEMIN1, suggesting that wound-induced STEMIN1 positively regulates their expression (Ishikawa *et al*., 2019). STEMIN2 and/or STEMIN3 may also activate these genes. The remaining 39 genes may be regulated by STEMIN1, STEMIN2 and/or STEMIN3.

(b) Conversely, 108 genes are downregulated in reprogramming leaf cells in a STEMIN-dependent manner. Notably, 11 genes of these genes have been previously shown to be upregulated by ectopic STEMIN1 expression (Ishikawa *et al*., 2019), suggesting that wound-induced STEMIN2 and/or STEMIN3 function to supress both the STEMIN1-mediated and STEMIN-independent pathways that activate these genes. The remaining 97 genes may be positively regulated by an unidentified wound-responsive pathway, which is suppressed by STEMIN1, STEMIN2 and/or STEMIN3.

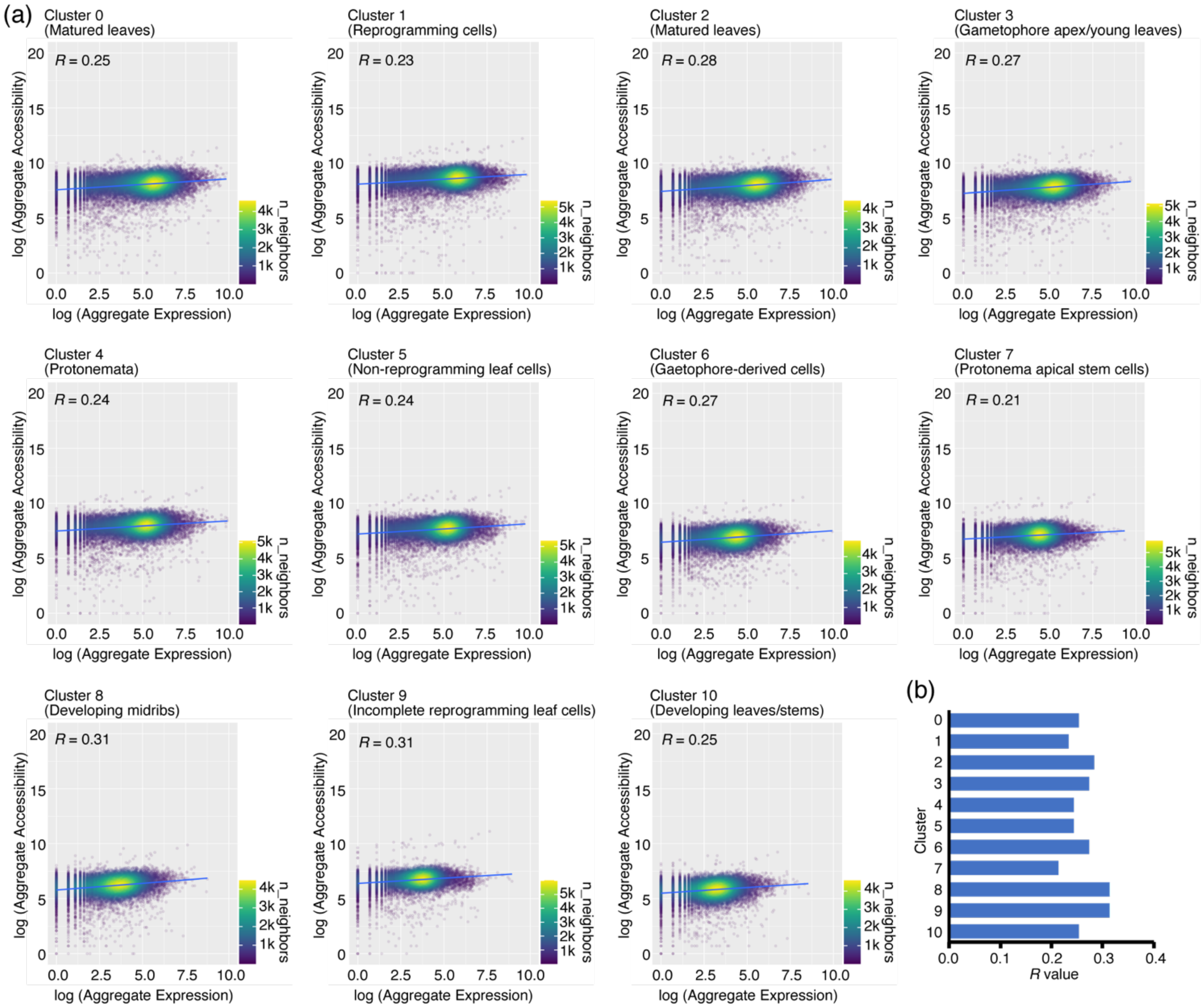

Figure S10. Correlation between gene expression and chromatin accessibility of all genes expressed in each cluster.

(a) Scatter plots illustrating the correlation between gene expression and chromatin accessibility for all genes (26,964 genes) within each cluster, as determined by Spearman's correlation coefficients. All presented correlations are statistically significant ($p < 0.001$). Colour indicates the density as number of nearest neighbours.

(b) Comparison of Spearman's correlation coefficients between clusters.

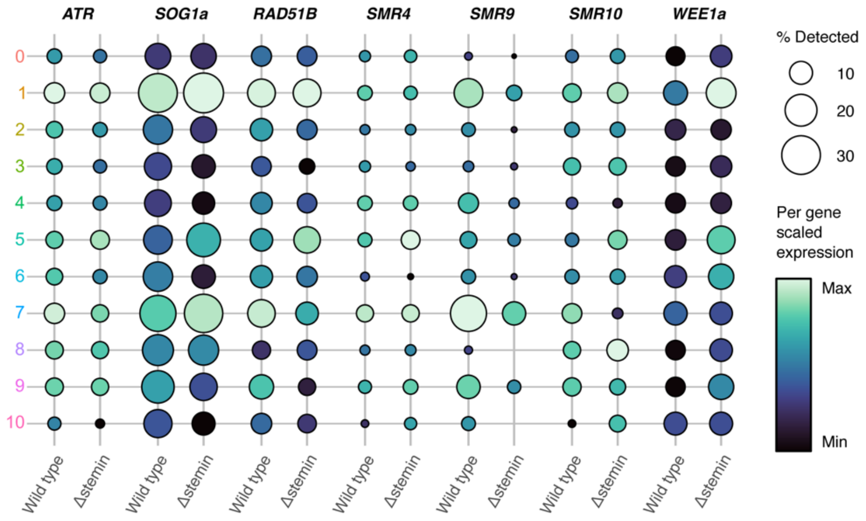

Figure S11. Dot-plots of genes involved in DNA damage response/repair in each cluster of wild-type and Δstemin plants.

Dot diameters and colours denote the proportion of cells and the average expression levels of the DNA-damage response and repair genes, respectively. Expression is scaled to the minimum and maximum cluster averages per gene of interest. See also the text.
